# Assessing MR-compatibility of somatosensory stimulation devices: A systematic review on testing methodologies

**DOI:** 10.3389/fnins.2023.1071749

**Published:** 2023-01-26

**Authors:** Carolina Travassos, Alexandre Sayal, Bruno Direito, João Pereira, Teresa Sousa, Miguel Castelo-Branco

**Affiliations:** ^1^Coimbra Institute for Biomedical Imaging and Translational Research (CIBIT), University of Coimbra (UC), Coimbra, Portugal; ^2^Siemens Healthineers AG, Lisbon, Portugal; ^3^Institute for Nuclear Sciences Applied to Health (ICNAS), University of Coimbra (UC), Coimbra, Portugal; ^4^Instituto do Ambiente, Tecnologia e Vida (IATV), Coimbra, Portugal; ^5^Faculty of Medicine (FMUC), University of Coimbra (UC), Coimbra, Portugal

**Keywords:** magnetic resonance imaging (MRI), functional MRI (fMRI), somatosensory stimulation devices, compatibility, safety, MR-compatible, MR-safe

## Abstract

Functional magnetic resonance imaging (fMRI) has been extensively used as a tool to map the brain processes related to somatosensory stimulation. This mapping includes the localization of task-related brain activation and the characterization of brain activity dynamics and neural circuitries related to the processing of somatosensory information. However, the magnetic resonance (MR) environment presents unique challenges regarding participant and equipment safety and compatibility. This study aims to systematically review and analyze the state-of-the-art methodologies to assess the safety and compatibility of somatosensory stimulation devices in the MR environment. A literature search, following the Preferred Reporting Items for Systematic Reviews and Meta-Analyses (PRISMA) statement guidelines, was performed in PubMed, Scopus, and Web of Science to find original research on the development and testing of devices for somatosensory stimulation in the MR environment. Nineteen records that complied with the inclusion and eligibility criteria were considered. The findings are discussed in the context of the existing international standards available for the safety and compatibility assessment of devices intended to be used in the MR environment. In sum, the results provided evidence for a lack of uniformity in the applied testing methodologies, as well as an in-depth presentation of the testing methodologies and results. Lastly, we suggest an assessment methodology (safety, compatibility, performance, and user acceptability) that can be applied to devices intended to be used in the MR environment.

**Systematic review registration:**

https://www.crd.york.ac.uk/prospero/, identifier CRD42021257838.

## 1. Introduction

Functional magnetic resonance imaging (fMRI) has established itself as a standard tool in human brain research due to its non-invasive character and high spatial resolution (Schimitt et al., 1998; Glover, 2011). The study of somatosensory functions through fMRI allows for the characterization of the brain-related areas, activity dynamics, and neural circuitries related to the processing of somatosensory information (Servos et al., 1998; Sanchez-Panchuelo et al., 2010; Kim et al., 2017; Kikkert et al., 2021).

The design of experimental paradigms that elicit activation of the somatosensory cortex requires the stimulation of a specific part of the body using either manual stimulation or mechatronic stimulation devices. Such devices can be used to study tactile perception, somatosensory processing, and effects of higher-order cognitive processes (e.g., attention) in somatosensory processing, while ensuring stimulation reproducibility, customization, and control (Gassert et al., 2006b). Moreover, they replace the manual application of the stimuli, which is prone to human error and impairs reproducibility. Piezoelectric-, pneumatic-, electromagnetic-, and electric-based devices are the most common types of somatosensory stimulation devices (Gassert et al., 2006b).

The development of mechatronic stimulation devices is hampered by the strict characteristics of the magnetic resonance (MR) environment.^1^ The MR environment is characterized by a strong static magnetic field (B0), switching gradients, and radiofrequency (RF) pulses which, together, enforce stringent restrictions on the material and actuation principles for any device intended to be used in MRI/fMRI procedures (Schaefers and Melzer, 2006; Schaefers, 2008; Hartwig et al., 2017). Regarding safety issues, the B0 is the main cause for the induction of displacement forces and torques on magnetic materials that may cause unwanted device movements (taking the risk to become a projectile) (Delfino and Woods, 2016). RF pulses are the main cause of induced voltages but also heat the device (may cause burns in the participant) or the surrounding tissue (Delfino and Woods, 2016). Heating may also be caused by induced voltages originated from gradient fields (Winter et al., 2021). Additionally, compatibility issues between the device and the MR environment may cause the device to malfunction such that it fails to deliver the intended stimulation profile or it conveys unintended physiologic stimulation caused by induced voltages (Schaefers and Melzer, 2006); the presence of the stimulation device might also induce image artifacts that lead to misleading interpretations in the MR images (Delfino and Woods, 2016). For these reasons, any device intended to be used within this area should undergo safety and compatibility assessments.

### 1.1. Safety assessments

Regarding safety, ASTM International published standards for the assessment of displacement force (ASTM F2052-21, 2021), magnetic torque (ASTM F2213-17, 2017), and radiofrequency heating (ASTM F2182-20, 2020). The International Standard Organization (ISO/TS) and the National Electrical Manufacturers Association (NEMA) provide other suitable methodologies for safety assessments. ISO/TS 10974 applies specifically to the assessment of active implantable medical devices (i.e., implantable medical devices containing a power source) (ISO/TS 10974, 2018), and NEMA MS8 and MS10 are used to measure specific absorption rate (SAR): in any type of device (NEMA MS 10, 2010; NEMA MS 8, 2016). There are no formal safety standards for non-implanted somatosensory stimulation devices. Nevertheless, some guidance can be drawn from the standards above.

### 1.2. Compatibility assessments

Concerning compatibility, artifacts can be introduced into the images if the device alters the homogeneity of the magnetic field. Special attention should be taken to fMRI acquisition sequences since they are particularly prone to susceptibility artifacts as a result of B0 field inhomogeneity (Abreu and Duarte, 2021) and scanner instability during longer exam sessions (Friedman and Glover, 2006). Additionally, the device performance should also be assessed according to each principle of actuation. Therefore, for a device to be considered MR-compatible, it is necessary to demonstrate that, in addition to safety, it performs as intended (without degradation on its own or MR-scanner functions) and hence does not cause image artifacts (ASTM F2503-20, 2020). ASTM F2119 reports a methodology to quantify image artifacts produced under a set of scanning conditions (ASTM F2119-13, 2013). NEMA MS3 and MS1 are suitable to determine image quality, namely image uniformity and signal-to-noise ratio (SNR), respectively (NEMA MS 1, 2020; NEMA MS 3, 2020).

### 1.3. Current standards

For research purposes, the aforementioned standards are not mandatory, prevailing the principles of Good Clinical Research Practice (such as the Helsinki Declaration and Good Clinical Principles) and study approval by local ethics committees or other responsible entities (World Medical Association [WMA], 2013; ISO 14155:2020, 2020). In opposition, for mechatronic stimulation devices intended for medical purposes, aside from the safety and compatibility assessments, a formal certification procedure must also be performed. According to the Food and Drug Administration (FDA) (U.S. Food and Drug Administration, 2021) and the standard for marking medical devices for safety in the MR environment from the ASTM International (ASTM F2503-20, 2020), a device can be labeled as MR-safe, MR-conditional, or MR-unsafe. By definition, MR-safe devices do not contain any metal and are composed entirely of materials that are electrically non-conductive, non-metallic, and non-magnetic; for this reason, an MR Safe medical device can safely be taken into any MR environment, without any additional safety risk to the patient, MR staff, and MR scanner. When a device presents additional risk to the subject or remaining individuals, it is labeled MR-unsafe. Additionally, a device is labeled MR-conditional if it poses no known hazards in a specific MR environment with specific conditions (that should be specified) but its behavior in other MR environments is not guaranteed (e.g., some devices are suitable to be in a 3T environment but not in a 7T).

Supplementary Table 1 summarizes the main international standards that apply to any medical device to be used in the MR environment, including somatosensory stimulation devices. We would like to emphasize that these standards were designed only to consider tests for the safety assessment of medical devices in the MR environment and artifacts in MR images, but do not relate specifically to fMRI procedures. Additionally, some of these standards apply to medical devices considered passive implants (i.e., devices that do not contain a power source and are partially/fully implanted). Additional precautions should be taken for active devices.

### 1.4. Objectives of this review

Here, we systematically reviewed the reports describing the development and safety/compatibility assessment of devices used for research purposes regarding the somatosensory stimulation of human participants in the MR environment. Additionally, we compile the most important tests applied by the records included in this review and the several standards on these matters in a practical, free-to-use protocol. In sum, the major goals of this report are:

• To present an overview of the current methodologies/practices for the design, development, and assessment of safety and compatibility of somatosensory stimulation devices intended for research purposes in the MR environment and discuss it based on the current international standards;
• To propose a complete assessment protocol for somatosensory stimulation devices to be used in the MR environment considering both the international standards and the current practices described in the reports included in this review.

This work primarily targets researchers working on the development of somatosensory stimulation devices. Commercial product development teams and technical leadership of MRI brain research facilities can also take advantage of this work.

## 2. Methods

### 2.1. Systematic review

To identify the studies that have developed and tested somatosensory stimulation devices in the MR environment we performed a systematic literature search following the guidelines defined in Preferred Reporting Items for Systematic Reviews and Meta-Analysis (PRISMA) (Page et al., 2021). According to these guidelines, the review protocol comprises four stages: identification, screening, eligibility, and inclusion.

#### 2.1.1. Identification

The search for relevant articles was done by CT in PubMed,^2^ Scopus,^3^ and Web of Science,^4^ according to the search terms in Table 1 (differences between search strings are due to the inherent characteristics of each database). The search reported here includes articles published up to November 2021 (literature search started in July 2021). Additionally, the references of the papers that were selected for full-text eligibility assessment were screened to retrieve further relevant publications.

**TABLE 1:** Search terms used in each of the chosen databases.

Database	Search strings
PubMed	(“magnetic resonance” OR “mr” OR “mr-” OR “functional magnetic resonance” OR “fMR” OR “fMR-” OR “functional magnetic resonance imaging” OR “fMRI” OR “fMRI-”) AND (“somatosensory” OR “somatotopic” OR “touch” OR “tactile”) AND (“compatible” OR “compatibility” OR “MR-compatib*” OR “safe” OR “safety” OR “MR-saf*”) AND (“design” OR “development”)
Scopus	[TITLE-ABS-KEY (“magnetic resonance” OR “mr” OR “mr-” OR “functional magnetic resonance” OR “fMR” OR “fMR-” OR “functional magnetic resonance imaging” OR “fMRI” OR “fMRI-”)] AND [TITLE-ABS-KEY (“somatosensory” OR “somatotopic” OR “touch” OR “tactile”)] AND [TITLE-ABS-KEY (“compatible” OR “compatibility” OR “MR-compatib*” OR “safe” OR “safety” OR “MR-saf*”)] AND [TITLE-ABS-KEY (“design” OR “development”)]
Web of Science	ALL = [(“magnetic resonance” OR “mr” OR “mr-” OR “functional magnetic resonance” OR “fMR” OR “fMR-” OR “functional magnetic resonance imaging” OR “fMRI” OR “fMRI-”) AND (“somatosensory” OR “somatotopic” OR “touch” OR “tactile”) AND (“compatible” OR “compatibility” OR “MR-compatib*” OR “safe” OR “safety” OR “MR-saf*”) AND (“design” OR “development”)]

#### 2.1.2. Screening

CT screened the title and abstract of the records returned in the identification phase. Studies of somatosensory stimulation devices following the inclusion criteria stated in Table 2 (“Study selection”) were considered. CT, AS, BD, JP, and TS reviewed the papers selected to assess the fulfillment of the eligibility criteria stated in Table 2 (“Eligibility assessment”). Each full text was independently reviewed by three authors, who voted for the inclusion of each paper. A paper was included if it had two or more votes, reducing the risk of bias. We included studies that described the development and assessment of devices used for somatosensory stimulation in the MR environment, whose tests were performed in phantom and/or adult human participants. We excluded studies with unclear descriptions of the testing protocols. In addition, we analyzed the reference list of selected records for additional relevant contributions which were also independently reviewed by three authors (following the procedure described above).

**TABLE 2:** Criteria for study selection and eligibility assessment.

	Inclusion criteria	Exclusion criteria
Study selection	- Devices for somatosensory stimulation in the MR environment; - Directly address device development and assessment; - Involvement of phantom or human adult participants (animal studies were excluded); - No time or date restriction.	- Non-English language; - Non-original articles (pre-prints, review articles, book series, commentaries, editorials, reviews, thesis, conference proceedings, and insufficient abstracts available will be excluded); - Not available online (or provided by the authors).
Eligibility assessment	- Somatosensory stimulation devices intended for use in the MR environment.	- Non-addressing device development; - Non-addressing performed tests.

#### 2.1.3. Inclusion

Each reviewer summarized the full text of the included papers, using a data extraction table that was created for this review (Supplementary Table 2). In this table, we summarized the device typology, design, and its main application, the MR scanner used for testing purposes, and a description of the tests applied to assess safety, compatibility, device performance, and user acceptability.

### 2.2. PROSPERO registration

Details of the protocol for this systematic review were registered in the international prospective register of systematic reviews (PROSPERO) under the ID: CRD42021257838 (Travassos et al., 2021).

## 3. Results

The results section is organized into three parts: the first provides an overview of the study selection, including a summary of the evaluated characteristics; the second part explores and characterizes the devices used for somatosensory stimulation in the MR environment included in this review; the third part characterizes the methodologies and conditions for device assessment.

### 3.1. Study selection

**FIGURE 1:**
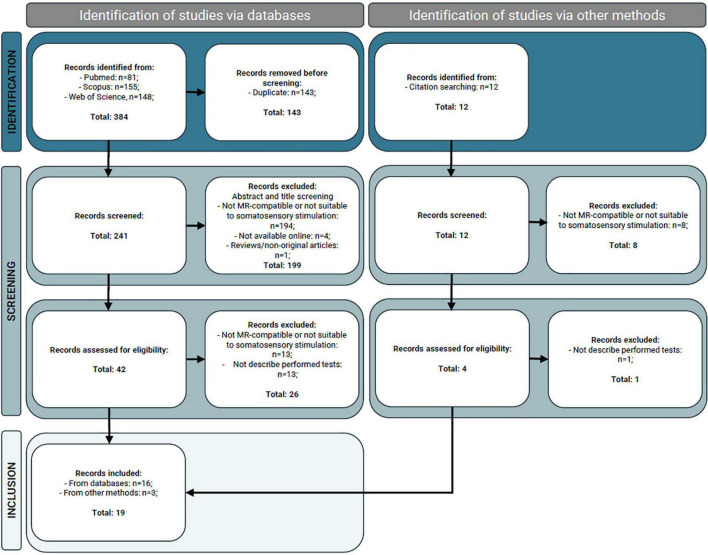
Preferred Reporting Items for Systematic Reviews and Meta-Analyses (PRISMA) 2020 flow diagram illustrating the magnetic resonance (MR) studies identification, screening, and inclusion (Page et al., 2021).

The search for relevant articles in databases identified 384 potential papers (81 identified via PubMed, 155 through Scopus, and 148 via Web of Science). From these, 143 duplicated records and 199 records that did not meet the inclusion criteria were excluded, resulting in 42 papers for full-text assessment. Among these, 13 records did not describe MR-compatible devices or devices designed for somatosensory stimulation (i.e., designed and utilized to perform tasks related to somesthesia with the goal of mapping the cortical representation of the body in the somatosensory cortex using MRI); other 13 did not describe the performed tests. This results in the inclusion of 16 records from databases research. Then, three additional articles were added from reference list screening. In the end, 19 records were included in this review. A flowchart illustrating the selection scheme is provided in Figure 1.

From the records included, we retrieved information about device typology, design, intended application, safety, compatibility, and other tests performed (including device performance and user acceptability), and MR scanners and sequences used for these assessments. This information can be found in Supplementary Table 2.

### 3.2. Characterization of the devices used for somatosensory stimulation in the MR environment

In this section, we provide a description of the technologies and components used in the design and development of somatosensory stimulation devices intended to be used in the MR environment, as well as the setup establishing communication between the control and the scanner room and the intended applications of described stimulation devices.

#### 3.2.1. Actuation principles

**FIGURE 2:**
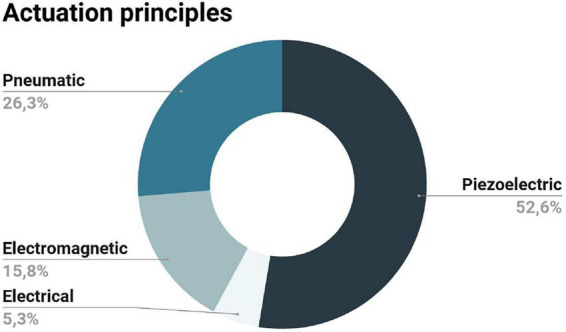
Actuation principles presented in the records included in this review: the majority of the records opted for the piezoelectric principle, followed by the pneumatic and the electromagnetic; the electrical-actuation method was described in a single record.

An appropriate choice of actuation technology is key in the development of mechatronic devices working within an MR environment. This choice considers the safety and compatibility constraints imposed by the high magnetic field, the switching gradients, and RF pulses. Here, we discuss the most common actuation principles based on the records included in this review and highlight some studies as examples. Figure 2 illustrates the actuation principles of the records included in this review.

##### 3.2.1.1. Piezoelectric

Piezoelectric devices are based on the piezoelectric effect, i.e., the capacity of a material to produce an electric charge when subjected to mechanical stress (Harrington et al., 2000). This effect is reversible, meaning that an applied voltage on a piezoelectric material will cause a mechanical strain (Harrington et al., 2000). This property, together with the fact that these are non-magnetic, makes piezoelectric devices strong candidates for use in the MR environment. In practice, these devices are able to deliver high frequency vibrotactile stimulation at a chosen amplitude (depending on the piezoelectric material and the applied voltage) (Kikkert et al., 2021). The main challenge with some piezoelectric materials is the need to apply a high voltage to get the required mechanical displacement (Harrington et al., 2000).

The majority of the records included in this review present somatosensory stimulation devices based on the piezoelectric effect (Harrington et al., 2000; Gassert et al., 2006a; Di Diodato et al., 2007; Yang et al., 2009, 2011; Duenas et al., 2011; Yu et al., 2011b; Guo et al., 2012; Chen et al., 2014; Wang et al., 2015). We would like to highlight the work of Harrington et al. (2000), which presented a vibrotactile stimulator made of piezoceramics (non-magnetic materials composed of special crystalline materials that are piezoelectric). The authors used high voltage batteries and an operational amplifier (which stayed in the console room) to generate the displacement of the piezoceramic material. The vibrotactile stimulator was designed and tested (for safety) with a small magnet outside the MR environment; as the device passed this test, it was then used in functional imaging tests. The authors proved its safety and effectiveness in the fMRI experiments performed. In another subject, a force feedback device for Virtual Reality fMRI made of a piezoelectric motor was developed (Di Diodato et al., 2007). The motor consisted of a stator (a small bar of aluminum with a piezoelectric crystal) and a rotor (pre-loaded by a string from the stator which transmits the oscillations and pushes the rotor around its axle). The authors considered the device MR-compatible and used it in 20 participants without incidents or image artifacts. Last example, the work of Gassert et al. (2006a) compared different approaches to constructing an fMRI-compatible mechatronic device with two degrees of freedom (DOF): a DC motor and an ultrasonic motor, concluding that the last one (based on the piezoelectric effect) was the best alternative to power the rotary degree of freedom of the device.

##### 3.2.1.2. Pneumatic

Pneumatic devices are mainly based on an air-puff technique (Huang et al., 2017). With these, it is possible to achieve strong stimulation intensities and they are suitable to stimulate any location on the human body, without significant restrictions on material selection (Huang et al., 2017; Kikkert et al., 2021). However, they may suffer from limited stimulation frequency ranges (due to the open pneumatic tube), low spatial resolution, and poor intensity control (Briggs et al., 2004; Kim et al., 2013).

Several somatosensory stimulation devices for MR applications included in this review have taken advantage of this actuation principle (Dresel et al., 2008; Montant et al., 2009; Hao et al., 2013; Goossens et al., 2016; Huang et al., 2017). The device developed by Hao et al. (2013) was capable of applying relatively high-pressure stimuli with a highly customizable programmable waveform and adjustable surface area. It was composed of an air compressor, a control unit, and an aluminum pneumatic actuator attached to a non-ferromagnetic platform where the feet rest (the only components inside the scanner room). Another study used von Frey-filaments (monofilaments made of acrylic glass which exert a constant, logarithmically scaled force) to apply point-like tactile stimuli in a precise spatiotemporal sequence to the face and hands of the subject; these applicators were controlled by magnetic valves that release the pressure from the pressurized air supply located outside the scanner room (Dresel et al., 2008).

##### 3.2.1.3. Electromagnetic

The electromagnetic method for somatosensory stimulation takes advantage of the strong and homogeneous static magnetic field of the MR scanner to generate vibratory stimuli (Kim et al., 2013). For this reason, electromagnetic devices are sensitive to placement and orientation inside the magnet (Briggs et al., 2004; Kim et al., 2013). These low-cost devices are simple, small, and use low voltage (Kim et al., 2013). However, they typically have limited stimulation frequency, intensity, and time resolution; the winding coil, whose actuator is large and not expandable to multichannel stimuli, also limits its applications (Kim et al., 2013). Some of these disadvantages were overtaken in the work of Kim et al. (2013) by developing an MR-compatible vibrotactile stimulator using a microcontroller (for precise control of the stimulation parameters), and planar technology (to make a high-quality planar-coil-type actuator). The actuator was based on the coiling of the printed circuit board (PCB) (instead of the conventional winding of copper wire on a bobbin), enabling “excellent repeatability and thermal characteristics, durability, humidity resistance, ability to assume various shapes, and lightweight construction.” They also designed a filter trap to protect the drive unit of the system from induced currents (due to the RF pulses and switching gradients). The works of Gallasch et al. (2006) and Carr et al. (2013) were also based on this principle of actuation.

##### 3.2.1.4. Electric

Stimulation devices based on electrical principles induce a tactile sensation using an electric current flowing through the skin, via electrodes placed on the skin surface (Hartwig et al., 2006). These devices have been used extensively outside of the MR environment to study the role of each type of mechanoreceptor and characterize their functional properties (Hartwig et al., 2006). The design of these devices implies additional precautions due to the high voltages involved (Gassert et al., 2006b). Since the functioning of electric devices is mainly based on conductive elements (such as cabling or metal structures), it is influenced by the magnetic field gradients and RF pulses (Gassert et al., 2006b). For this type of device, the only report included in this review opted to place inside the scanner room only the stimulator pad (made of plexiglass and aluminum), leaving outside the main electronic components (Hartwig et al., 2006).

#### 3.2.2. Communication between the control and MR scanner rooms

**FIGURE 3:**
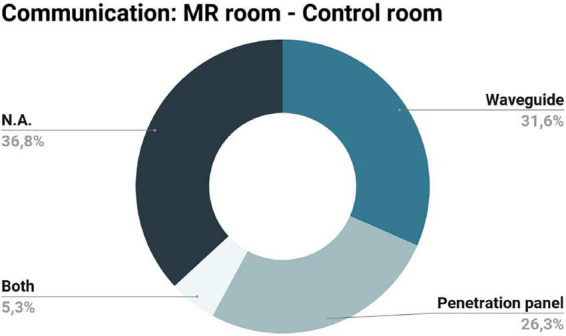
Communication methods between the scanner and the control rooms described in the records included in this review: a similar percentage of records opted for the waveguide and penetration panel and only one paper used both configurations; the remaining records did not specify the communication method (N.A.).

All the devices presented in the records included had the control system (electronics) and the power sources located outside the scanner room. Communication between the scanner and the control rooms is possible through the waveguide or the penetration panel (using RF filters to keep the Faraday cage intact and keeping the scanner room shielded from electromagnetic interference). Figure 3 summarizes the communication methods utilized by the records included in this review. The penetration panel, together with RF filters, was used by the following papers: (Harrington et al., 2000; Di Diodato et al., 2007; Dresel et al., 2008; Yang et al., 2011; Wang et al., 2015). On other hand, the following studies opted for the waveguide: (Gassert et al., 2006a; Hartwig et al., 2006; Carr et al., 2013; Hao et al., 2013; Kim et al., 2013; Huang et al., 2017). The remaining papers do not explicitly report this issue (Gallasch et al., 2006; Montant et al., 2009; Yang et al., 2009; Yu et al., 2011b; Guo et al., 2012; Chen et al., 2014; Goossens et al., 2016), excepting the work of Duenas et al. (2011), where (according to our interpretation) both communication methods were used (the penetration panel, with RF filters, for electrical power and data transmission, and the waveguide for fiber optical switches).

#### 3.2.3. Stimulation intended applications

**FIGURE 4:**
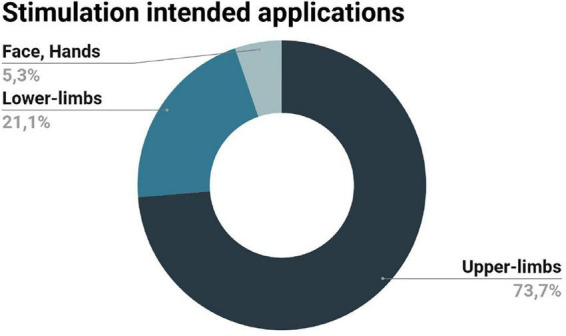
Intended applications of the stimulation devices described in the records included in this review: stimulation of the upper limbs was the main application of the devices considered here, followed by the stimulation of the lower limbs, the face and the hands.

The majority of the records included in this review were intended for upper-limb somatosensory stimulation, mainly the fingers, which have a larger cortical representation in the primary somatosensory cortex (S1) (Harrington et al., 2000; Gassert et al., 2006a; Hartwig et al., 2006; Di Diodato et al., 2007; Montant et al., 2009; Yang et al., 2009, 2011; Yu et al., 2011b; Guo et al., 2012; Carr et al., 2013; Kim et al., 2013; Chen et al., 2014; Wang et al., 2015; Huang et al., 2017). Only one study was dedicated to the development of a computer-controlled MR-compatible stimulation device for punctate tactile stimuli of the face and hands (Dresel et al., 2008). The remaining studies were dedicated to the development of stimulation devices for lower-limb stimulation (Gallasch et al., 2006; Duenas et al., 2011; Hao et al., 2013; Goossens et al., 2016). Figure 4 summarizes the stimulation intended applications of the included records.

### 3.3. Characterization of the methodologies and conditions for device assessment

Designing devices to work within the MR environment requires the fulfillment of a number of safety and compatibility criteria. In this section, we provide a comprehensive overview of the main assessment methodologies that the records included in this review applied.

#### 3.3.1. Safety tests

**FIGURE 5:**
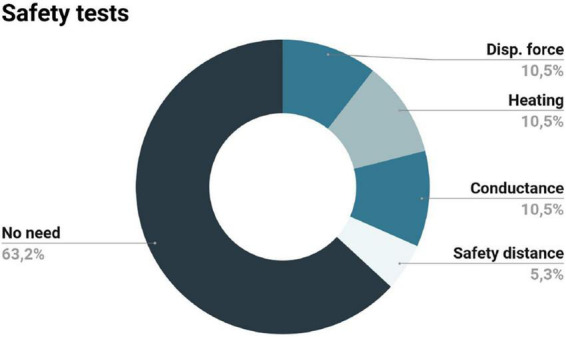
Safety tests applied to the stimulation devices considered in the records included in this review: the majority of the records included did not need to perform a safety assessment; of those who did it, two evaluated the displacement force (Disp. force), the conductance between leads, and the heating of the device; only one record defined the safety distance for device safe-operation.

Safety assessment determines if the presence of the device in the scanner room may cause injury to the participants or MR technologists and damage to the MR scanner itself. Safety tests assess devices made of inherently MR-unsafe materials. Of the nineteen records included in this review, three considered the safety assessment of their devices; Figure 5 summarizes this information, including the safety tests performed.

In the study of Wang et al. (2015), a piezoelectric vibrotactile system was developed to stimulate participants’ hands. The authors performed safety tests on the components to be located inside the scanner room (piezoelectric elements and the Bayonet Neill-Concelman coaxial wires). First, an initial inspection to detect any pull toward a strong magnet was done outside the MR scanner room. The authors verified that the cable connector was affected by the magnet, however, since the connector is positioned at the interface panel (located outside the 10-gauss line), they did not consider it a major concern. Then, the cable and the vibrotactile system were carefully brought inside the scanner room and a multimeter was used to confirm negligible conductance between the two electrical leads on the stimulation device (eddy currents). A temperature probe was also used on the device while the MR scanner was operating to confirm negligible heating. The authors concluded that the device and its constituents were safe to use in the MR environment.

The work of Gallasch et al. (2006) consisted in the development of an electromagnetic stimulation device to study the cerebral responses to foot stimulation. Since the vibration probes were ferromagnetic, the authors highlighted the need for safety and compatibility assessment. Regarding safety, they used a Gaussmeter to determine the minimal distance for the safe operation of the device, corresponding to the 20 mT line. Moreover, they added brackets with screws to clamp the device to the scanner sled and highlighted the need for constant vigilance to ensure that the device is not inadvertently moved too close to the magnet. The authors also reported minimal effects on imaging and performance (when the device operates behind the 20 mT line).

The last study that performed safety tests was from Harrington et al. (2000) (although the authors have called them compatibility tests). They evaluated the attraction force (using a small permanent magnet over the piezoceramic wafer) and the heating of the wire of their piezoelectric device on a 1.5T MRI setup. The authors concluded that the small magnet did not produce a displacement force in the piezoelectric wafer and no noticeable increase in the temperature of the wire was noticed. The wafer was considered MR compatible, and it was used by the subject (in their hands) while in the scanner. The wire was kept as far away from the scanner as possible, running parallel to the bore of the magnet and a lower input current was used (to reduce the induced force caused by the interactions between the magnetic field of the scanner and the current in the coaxial wire–according to Faraday’s law).

The remaining records did not perform a safety assessment since the electronic and/or ferromagnetic components were located outside the scanner room.

#### 3.3.2. Compatibility tests

**FIGURE 6:**
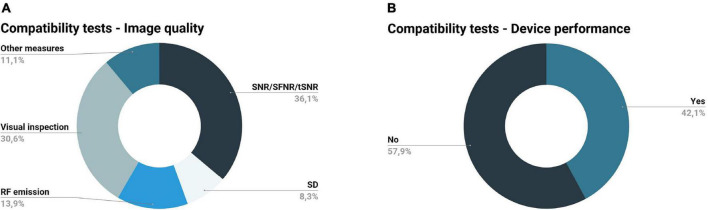
Compatibility tests performed by the records included in this review. **(A)** Image quality metrics: signal-to-noise ratio (SNR), temporal signal-to-noise ratio (tSNR), and signal-to-fluctuation-noise (SFNR) were the most utilized metrics to evaluate image quality, followed by visual inspection, radiofrequency (RF) emission, standard deviation of the image intensity time-course (SD), and power spectral density; some records did not specify the metrics assessed (other measures). **(B)** Device performance evaluation: the majority of the records did not verify the device performance in the magnetic resonance (MR) environment.

Compatibility tests are designed to verify whether a device does not adversely affect the quality of the images being acquired and its operation is not affected by the scanner (ASTM F2503-20, 2020). A device is considered MR-compatible if both conditions are satisfied (ASTM F2503-20, 2020). With the exception of the work of Harrington et al. (2000) (which described the performed safety tests as compatibility tests without performing additional compatibility tests as usually defined), all studies reported the compatibility tests performed. Figure 6 summarizes the compatibility tests concerning image quality (Figure 6A) and device performance (Figure 6B) described in the records included.

The more utilized measures to evaluate image quality were related to the SNR, temporal signal-to-noise ratio (tSNR), and signal-to-fluctuation-noise (SFNR) (Gassert et al., 2006a; Hartwig et al., 2006; Di Diodato et al., 2007; Montant et al., 2009; Yang et al., 2009, 2011; Duenas et al., 2011; Guo et al., 2012; Carr et al., 2013; Hao et al., 2013; Kim et al., 2013; Chen et al., 2014; Huang et al., 2017) (Figure 6A). These metrics are useful to examine the quality of structural (SNR) and functional (tSNR, SFNR) images. SNR compares the signal of the MR images to the background noise of the image, while tSNR and SFNR are both metrics for the temporal quality; the first is defined as the ratio between the mean signal intensity over time and its temporal standard deviation, and the second is defined as the mean intensity divided by the standard deviation of the total noise (Greve et al., 2011; Jarrahi and Mackey, 2018; Lu et al., 2019). In most cases considered in this review, these metrics were accompanied by a visual inspection of the images (Gallasch et al., 2006; Hartwig et al., 2006; Di Diodato et al., 2007; Dresel et al., 2008; Duenas et al., 2011; Yu et al., 2011b; Carr et al., 2013; Hao et al., 2013; Kim et al., 2013; Chen et al., 2014; Goossens et al., 2016).

Although quality measures and statistical comparison of compatibility metrics between testing conditions were described in most of the studies included, the only reference to NEMA standards was made in the work of Hao et al. (2013) to calculate the SNR. Another study described in detail statistical tests used to evaluate changes in SNR and time-domain standard deviation performed in two stages of the device development (Gassert et al., 2006a); based on the results of these tests, the authors were able to choose the components with the best properties for the selected application, guaranteeing MR compatibility.

Overall, there were three main testing conditions: (1) no device in the scanner room (reference condition); (2) device in the scanner room (inside or outside the bore) but not in operation (silent device); (3) device in the scanner room, in operation (functioning device) (Gassert et al., 2006a; Hartwig et al., 2006; Di Diodato et al., 2007; Dresel et al., 2008; Carr et al., 2013; Hao et al., 2013; Kim et al., 2013; Wang et al., 2015; Goossens et al., 2016). All the records included used phantoms (simulating the human body) for compatibility assessments.

Still regarding compatibility assessment, it is also required to assess device performance in the MR environment (ASTM F2503-20, 2020). Such assessment corresponds to the verification of the device performance inside the MR scanner room, where it is subjected to B0, switching gradients, and RF pulses. The device performs as intended if its function is not affected by the MR environment. Eight out of the nineteen studies included in this review analyzed the device performance (Di Diodato et al., 2007; Montant et al., 2009; Yang et al., 2011; Yu et al., 2011b; Hao et al., 2013; Kim et al., 2013; Wang et al., 2015; Huang et al., 2017) (Figure 6B). The work of Wang et al. (2015) examined amplifier gain and voltage fed to a piezoelectric device in the MR environment, concluding that these measures were not affected by the functioning MR scanner; they also analyzed the vibration level inside and outside the scanner room, concluding that the vibration applied to the participant did not change with the MR acquisition. Another record reported the performance of stimulation force profile tests in the MR environment, i.e., check if the profile of stimuli force on the skin is in accordance with the theoretical stimulation parameters (Huang et al., 2017). In another study, the efficiency and reliability of a pneumatic device inside and outside the scanner were tested (Montant et al., 2009); the authors measured the vibration frequency as a function of the percentage of the aperture of the flowmetric gate (frequency calibration) and found that appropriate vibrations for activating muscle spindles were generated. However, the vibration frequency was slightly lower inside the scanner room, possibly due to the off-centered mass made of barite, which is slightly diamagnetic. The work of Kim et al. (2013) presented a complete assessment of the electromagnetic stimulation device, including the assessment of the performance of the filter trap, measurement of the stimulation signals inside the scanner, and investigation of the stimulation intensity changes due to frequency changes. Di Diodato et al. (2007) opted to compare the performance of their device with other non-fMRI compatible haptic devices, concluding that the prototype performs within the ideal and typical ranges of these devices. The work of Hao et al. (2013) studied the relation between the voltage input and the force output of the actuator inside the magnet room before testing it on the subject. Results indicated the existence of eddy currents under conditions of varying stimulation frequencies; the authors tested the potential effect of this on the force output inside and outside the scanner, concluding that the peak forces outside the magnet room are not affected by the frequency of oscillations but resulted in a small but significant reduction inside the magnet room. Previous research has already found it, so this should be taken into consideration when designing stimulation protocols (Jeneson et al., 2010). Lastly, Yang et al. (2011) and Yu et al. (2011b) stated the evaluation of the function, precision, and performance and the evaluation of the operational reliability and performance of their devices, respectively. In both studies, the results show that the system performance was not affected by the magnetic field.

#### 3.3.3. Other tests

**FIGURE 7:**
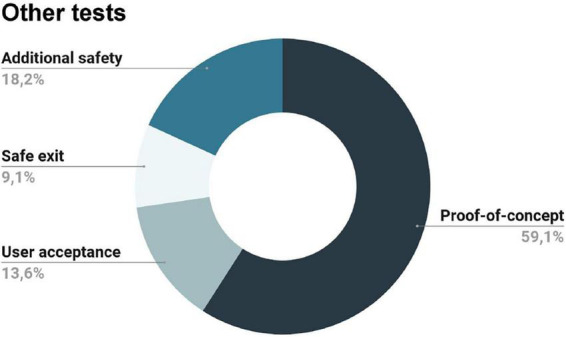
Additional tests performed by some of the included records: proof of concept imaging studies, additional safety measures, user acceptability tests, and evaluation of the participant’s safe exit from the magnetic resonance (MR) scanner in case of an emergency.

Besides safety and compatibility assessments, some studies performed additional tests (Figure 7). Proof-of-concept imaging studies (with one or few subjects), demonstrating the brain activation of the regions linked to somatosensory function, were performed by the following authors: (Harrington et al., 2000; Gallasch et al., 2006; Gassert et al., 2006a; Hartwig et al., 2006; Di Diodato et al., 2007; Dresel et al., 2008; Yang et al., 2009; Duenas et al., 2011; Carr et al., 2013; Hao et al., 2013; Kim et al., 2013; Goossens et al., 2016; Huang et al., 2017).

Additionally, some papers also reported the user acceptability of the stimulation device and the reported effects of the stimulation (Harrington et al., 2000; Hartwig et al., 2006; Wang et al., 2015). In this category, we only considered reports that have explicitly assessed user acceptability. For instance, a study applied a behavioral questionnaire at the end of the fMRI acquisition to find out whether the perceived intensity of stimulation changed significantly throughout the experiment; however, this was done to keep subjects engaged in the task during the acquisition, so it was not considered (Dresel et al., 2008).

Lastly, only two studies considered the consequences of the presence of the device in case of the safe exit of the subject in an emergency (Yang et al., 2009, 2011). According to the authors, their devices did not preclude the safe exit of the subject in an emergency; besides, they also had a safety system that stopped all operations if needed and a plastic frame containing the control cables and drive motors (preventing contact with the subject). Other records also mentioned additional safety measures, such as the existence of a safety button to stop all operations in case of an emergency or additional software safety routines (Gassert et al., 2006a; Duenas et al., 2011; Yu et al., 2011b; Guo et al., 2012).

#### 3.3.4. MR scanners and acquisition sequences

**FIGURE 8:**
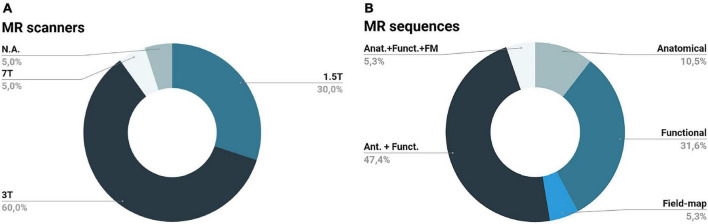
Magnetic resonance scanners **(A)** and sequences **(B)** utilized by the records included in this review: the majority of the records tested their devices at a 3T scanner (some records did not specify the scanner utilized (N.A.) utilizing both anatomical (Ant.) and functional (Funct.) acquisition sequences; remaining records used functional, anatomical, field-map (FM) or a combination of all these sequences.

The majority of the records tested the stimulation devices on 3T (Di Diodato et al., 2007; Montant et al., 2009; Yang et al., 2009; Yu et al., 2011b; Guo et al., 2012; Carr et al., 2013; Hao et al., 2013; Kim et al., 2013; Chen et al., 2014; Goossens et al., 2016; Huang et al., 2017) MR scanners; only one study tested both on 3T (Siemens Trio) and 7T (Siemens Magnetom) MR scanners (Duenas et al., 2011) and another did not specified (Wang et al., 2015); the remaining studies tested on 1.5T MR scanners (Harrington et al., 2000; Gallasch et al., 2006; Gassert et al., 2006a; Hartwig et al., 2006; Dresel et al., 2008; Yang et al., 2011). Figure 8A summarizes the previous information.

Regarding the MR sequences utilized, Yang et al. (2011) and Wang et al. (2015) only used anatomical sequences. However, the majority of the studies tested the impact of the device both on anatomical and functional sequences (Gassert et al., 2006a; Hartwig et al., 2006; Di Diodato et al., 2007; Dresel et al., 2008; Yang et al., 2009; Yu et al., 2011b; Guo et al., 2012; Carr et al., 2013; Goossens et al., 2016). Some of the anatomical sequences utilized were: T1-weighted high-resolution, T1-weighted magnetization-prepared rapid acquisition with gradient echo (MPRAGE), 3D fast spoiled gradient echo sequence, Spoiled gradient echo, T1-weighted spoiled gradient echo image with inversion recovery, T1-weighted spoiled grass, and turbo field echo. Regarding functional sequences, the most used were: gradient echo-planar imaging (GE-EPI), T2-enhanced (T2*) EPI, spin-echo EPI (SE-EPI), and T2* GE-EPI. Some studies also employed field map sequences (Duenas et al., 2011) or field-map combined with anatomical and functional sequences (Hao et al., 2013). Figure 8B summarizes the previous information.

## 4. Discussion

This systematic review focuses on the development and assessment of somatosensory stimulation devices intended to be used in the MR environment, highlighting the stringent conditions of the MR environment and the consequent impact on the design, development, and assessment of somatosensory stimulation devices for research purposes. Several other studies, whose devices were not primarily designed for somatosensory stimulation, should be mentioned while not being primarily included in this review. Devices designed for haptic feedback interfaces and human-machine interaction also provide important contributions in this field since they also presented safety and compatibility assessments for their devices (Hribar et al., 2009; Wang et al., 2009; Su et al., 2012; Farkhatdinov et al., 2022).

To the best of our knowledge, there is only one review in this field focused on safety and compatibility tests for any mechatronic device intended to be used in fMRI experiments (Hartwig et al., 2017). The authors identified two major problems: (1) the absence of citation international standards that should guide device testing, and (2) the lack of standardized procedures to test devices. These are key aspects that we also identify in this work. The variability in the reporting of the studies in this research area limits the standardization of methodologies for the safety and compatibility assessment of novel stimulation devices, reinforcing the need for reviews such as this one. We discuss the main findings in the context of the currently available standards in the field and finish proposing an MR-compatibility assessment protocol based on the current methodologies and the international standards.

### 4.1. Design and development of somatosensory stimulation devices to be used in the MR environment

An easier solution when designing a stimulation device for MRI/fMRI is to opt for components made of MR-safe materials. This would ensure that it is safe to use such a device in the MR environment without inducing image artifacts. However, this is not always practical, depending on the type of device, intended application, and its actuation principle. A number of technical actuation principles have been explored in the studies included in this review, each with its own advantages and disadvantages. In summary:

• Pneumatic stimulation is probably the best choice concerning MR safety and compatibility since the hardware typically can be made of MR safe materials (e.g., plastic tubes) (Huang et al., 2017; Kikkert et al., 2021); besides, they can achieve strong stimulation intensities (Huang et al., 2017). However, they have a limited frequency range of vibration, low spatial resolution, poor intensity control, and long transient response times (Golaszewski et al., 2012; Kim et al., 2013; Hartwig et al., 2017; Huang et al., 2017);
• Electromagnetic stimulators are easy to manufacture and implement, being low-cost systems with a wide range of stimulation parameters (Golaszewski et al., 2012; Kim et al., 2013; Hartwig et al., 2017). On other hand, they are prone to artifacts due to the interaction with the magnetic field, as well as their stimulation intensity varies according to the position and orientation in the field; besides, they have limited stimulation frequency, intensity, and time resolution (Golaszewski et al., 2012; Kim et al., 2013; Hartwig et al., 2017);
• Piezoceramics/piezoelectrics, despite being capable of a wide range of frequencies and being easy to control, require a high voltage to generate relatively small stimuli (displacement amplitudes) and may contain electronic components and electric wires that may affect the MR signal (Golaszewski et al., 2012; Hartwig et al., 2017; Huang et al., 2017);
• Electrical stimulators are widely customizable but additional safety and compatibility precautions are needed since they are prone to safety and compatibility issues due to their complex architecture (Harrington et al., 2000; Gassert et al., 2006b; Hartwig et al., 2017).

**FIGURE 9:**
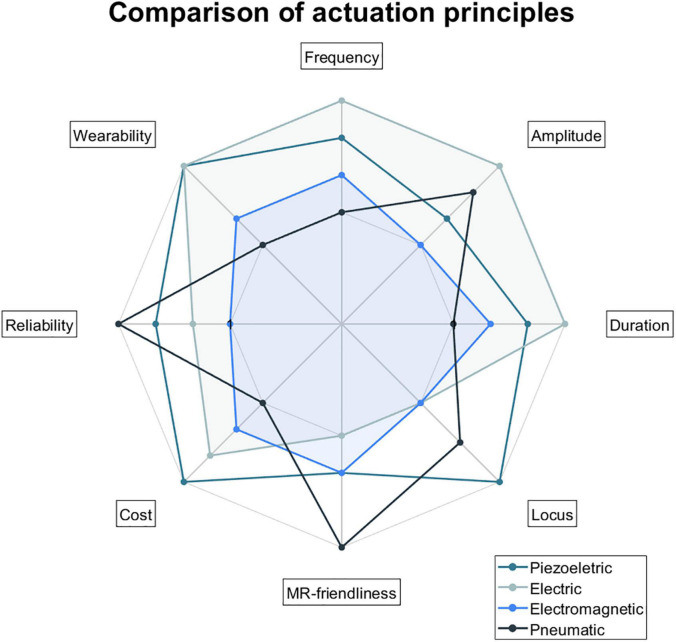
Comparison of actuation principles in the following categories: frequency, amplitude, duration, locus, MR-friendliness, cost, reliability, and wearability. Actuation principles were ranked based on the subjective opinion of the authors after considering the records included in this review and the general literature. A high ranking means a better position compared to the other actuation principles (frequency: wide range of frequencies; amplitude: high intensity of vibration; duration: better temporal variation in the stimuli presented; locus: small contact area/better spatial resolution; MR-friendliness: less ferromagnetic components; cost: cheaper; reliability: more reliable; wearability: more wearable).

Figure 9 aims to show the positioning of each principle of actuation in terms of the following categories: frequency, amplitude (i.e., the intensity of vibration), duration of vibration (i.e., temporal variation in the stimuli presented; involving burst duration, pulse repetition rate, and the number of pulses), locus (i.e., area of the contactor stimulating the skin), MR-friendliness (i.e., types of materials involved), cost (i.e., production cost), reliability, and wearability. These categories were decided based on the most important features for somatosensory stimulation in the MR environment identified by (Jones and Sarter, 2008). Actuation principles were ranked based on the subjective opinion of the authors after considering the records included in this review and the general literature. A high ranking means a better position compared to the other actuation principles (frequency: wide range of frequencies; amplitude: high intensity of vibration; duration: better temporal variation in the stimuli presented; locus: small contact area/better spatial resolution; MR-friendliness: less ferromagnetic components; cost: cheaper; reliability: more reliable; wearability: more wearable).

Lastly, none of the records included gave details regarding triggering methods, their timing delays, and possibilities of wireless interface options so these points were not considered in this work.

### 4.2. Methodologies and conditions for the assessment of somatosensory stimulation devices

#### 4.2.1. Safety assessment

Determine if the presence of the device may cause injury to the participants or MR technologists and damage the MR scanner itself.

Three of the included records performed safety tests on their devices (Harrington et al., 2000; Gallasch et al., 2006; Wang et al., 2015). The remaining records had the control mechanisms and the power source (containing MR unsafe components) of the proposed devices outside the MRI scanner room. This way, the authors argued that MR safety is guaranteed, and no safety tests are needed. Some cases might however deserve additional discussion. An example is the tactile stimulation device developed by Dresel et al. (2008). This device had a driving unit containing electromagnetic valves and double action-pneumatic cylinders positioned underneath the patient table (i.e., relatively close to the scanner bore) inside an electrically grounded aluminum case (acting as a Faraday cage). Possibly, more information would be required to exclude the need for safety tests. Another situation is the study where a cable connector affected by the magnet used at the initial safety assessment (outside the scanner room) is used in the scanner room (Wang et al., 2015). The authors argued that this is not a major concern since the connector is positioned at the interface panel (located outside the 10-gauss line); no additional precautions were taken except the recommendation of care during the installation of the connector.

#### 4.2.2. Compatibility assessment

Compatibility tests aim to verify whether a device, in addition to being safe, performs well in the MR environment and does not affect any measure of MR image quality (ASTM F2503-20, 2020). The most common assessment of compatibility is performed by visual inspection of phantom images. A visual inspection enables checking for the most common interference patterns in the data. Image subtraction also enables the identification of distortions and artifacts in the data. While the above is useful to look for degradation of image quality, temporal stability measures are crucial in the context of fMRI data, since the neural activity is inferred by modeling the signal changes across time (Hartwig et al., 2017). Temporal signal-to-noise (tSNR) measures are used to examine whether the deviation of the signal of a voxel over time is affected by any device used during imaging. For this purpose, one of the more useful measures is the standard deviation of the image intensity time-course (Gassert et al., 2006a; Hartwig et al., 2006; Hao et al., 2013).

In general, the MR sequences used for functional imaging are more sensitive to susceptibility artifacts (Abreu and Duarte, 2021). In this sense, the device should be tested using the sequences that will be applied in the future during real acquisitions with participants. Overall, the authors of the studies reviewed here report the MRI scanner and sequences used in their assessments.

A few records mentioned the assessment of device performance in the MR environment. Interactions between the device and the MR gradients may cause unintended physiological stimulation or device malfunction (Schaefers and Melzer, 2006). However, no appropriate standardized test method for non-implantable medical devices is yet available. Therefore, the assessments reported in the studies included in this review were highly inconsistent.

#### 4.2.3. Other tests

A few papers also considered participant acceptability. Due to the limited space inside the scanner bore, additional devices placed inside it may increase participant discomfort. In addition, the operation of the device (i.e., vibration) should not be uncomfortable for the participants. Lastly, some records mentioned the need for a safe exit of the participant in case of an emergency and additional safety measures. Stimulation devices with support platforms fixed to the scanner table (Hao et al., 2013) may impair the quick exit of the participant in case of need.

### 4.3. Proposal of MRI safety, compatibility, and user acceptability assessment protocol

**FIGURE 10:**
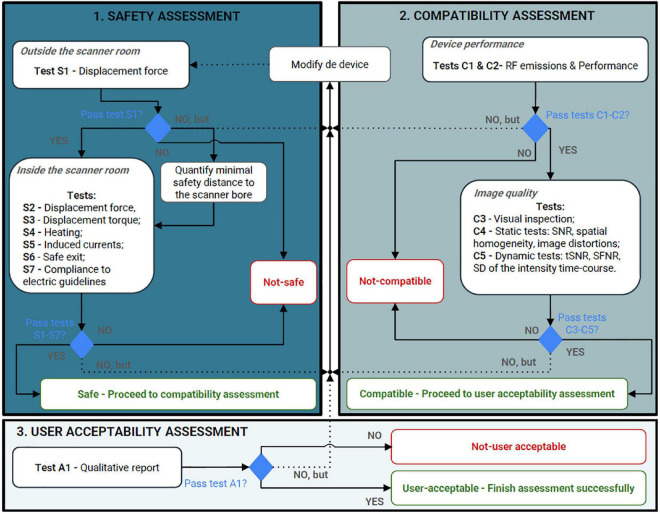
Proposed protocol to assess devices intended for research purposes to be used in the magnetic resonance environment according to the information found in our review. The assessment process should start from safety assessment, passing to compatibility (including device performance and image quality assessments), and finally, acceptability assessment. RF, radiofrequency; SNR, signal-to-noise ratio; tSNR, temporal signal-to-noise ratio; SFNR, signal-to-fluctuation-noise; SD, standard deviation.

Based on the analysis performed, the work of Hartwig et al. (2017), and the available standards, a complete assessment methodology to test the safety, compatibility, and user acceptability of somatosensory stimulation devices to be used in the MR environment for research purposes is presented here (Figure 10). The proposed testing pipeline includes three main stages: (1) safety assessment (to be conducted when the device’s components are ferromagnetic), (2) compatibility assessment (mandatory), and (3) user acceptability assessment (mandatory). Please note that we did not consider any specific procedure suggested by the MR scanner manufacturers. The presented assessment might be time/resource-consuming so authors are responsible for selecting the ones more urgent according to their device and intended application.

#### 4.3.1. Safety assessment

Safety considerations should be considered already in the design of the device; the stringent characteristics of the MR environment and the particular characteristics of the scanner where the device will be used constraint options in terms of materials and operating principles.

Safety tests must be done whenever the device has constituents that are not inherently MR-safe [for a deep discussion of the influence of magnetic susceptibility on the MR compatibility of materials, see (Schenck, 1996)].

Initially, a safety assessment outside of the scanner room using a permanent hand magnet (strong enough to best simulate the intensity of the static magnetic field where the device will be positioned) is advisable. Test and, whenever is the case, quantify the provoked displacement force and torque. To pass this test, there should be no force applied by the magnet to each component of the device. If this is not the case, researchers have two options: modify the device (and test it again) or determine the minimal safety distance to the scanner bore for device safe operation [as in the work of Wang et al. (2015)]. On other hand, if no forces/torque are noticed, the device can be tested in the scanner room.

Within the MR environment, required tests include the evaluation of the magnetically induced displacement force (ASTM F2052-21, 2021), magnetically induced torque (ASTM F2213-17, 2017), induced heating (ASTM F2182-20, 2020), and induced voltages (no available standard). ASTM F2052^5^ compares the magnetically induced force to the weight of the device; the acceptance criterion is “magnetic force less/equal than medical device weight/gravity force.” ASTM F2213^6^ compares the magnetically induced torque to the equivalent torque applied by the gravitational force; the acceptance criterion is “torque less/equal than gravitational torque.” Concerning heating, the standard test method from ASTM F2182 is specific for passive implants (ASTM F2182-20, 2020).^7^ However, there is no other international standard published for heating assessment in external devices. In any case, a careful assessment of temperature should be conducted. All devices should work according to their operating temperature and special attention should be taken when some of the device components are in contact with the participant. Induced voltages can drive currents that can cause unintended physiologic stimulation or device malfunction (Schaefers and Melzer, 2006); measurements of induced voltages are described in some publications–for details see Georgi et al. (2004), but an appropriate standardized test method is not yet available. We recommend checking for any induced-voltages with a phantom and an equivalent-setup to a human participant. Only after verification of the device’s correct functioning under all conditions, should the device be considered for the next evaluation steps of this protocol.

Lastly, we recommend considering emergency situation, such as the need to remove the participant from the scanner. The device should not prevent the safe exit of the participant; for this reason, fixed structures are not advised, and the device must be compact enough and capable of fitting inside the scanner bore (if necessary) without causing excessive discomfort to the subject during scanning. Additional safety systems to stop all operations if needed (e.g., safety button), as well as software routines to confirm the stimulation parameters are within the desirable range, are highly advisable.

In the case of electric-based devices, redundant safety measures should be included. IEC 60601-1 is the general standard applying to the basic safety and essential performance of medical electrical equipment/systems (IEC-60601-1:2005/AMD1:2012/ISH1:2021, 2021). It defines the general requirements and serves as the basis for other particular standards, such as IEC 60601-1-2 (focusing on electromagnetic compatibility) (IEC-60601-1-2:2014/AMD1:2020, 2020) and IEC 60601-2-10 (focusing on nerve and muscle stimulators) (IEC-60601-2-10, 2009). Specific safety measures for electric-based devices are listed next:

• Regarding stimulus pulse outputs: the maximum energy per pulse shall not exceed 300 mJ when applied to a load resistance of 500 ohms and the maximum output voltage shall not exceed a peak value of 500 V, when measured under open-circuit conditions (IEC-60601-2-10, 2009);
• Regarding power: low-power batteries should be used [such as in the work of Hartwig et al. (2006)];
• Regarding electrically conductive wires or leads: loops must be avoided to prevent induced currents and potential heating/burn of the skin of the participant; leads’ length must be appropriate (to avoid the establishment of a resonant circuitry between the RF energy and the wire/lead) and they must be properly shielded; attention should be paid to mechanical forces (such as Lorenz forces and resulting torques) caused by large electrical currents; to avoid serious consequences to the participant, thermal insulation (including air, pads, etc.) between the patient and the electrically conductive material can be used as additional precaution as well as keep the leads/wires as far as possible from the patient (ACR Committee on MR Safety, 2020);
• Regarding software: additional emergency and fault detection routines surveilling device functioning should be implemented;
• Regarding electrodes: never place the stimulation electrodes on opposite sides of the body nor in a way that stimulation currents are allowed to flow through the subject’s heart; place the electrodes as far away from the heart as possible and put the positive and negative electrodes closely together; to diminish the skin irritation, choose electrodes with lower salt content; lastly, electrode leads must be axially twisted from cable junction to stimulation site to reduce conductive loop cross-sectional area and must be as short as possible (BIOPAC, 2022a,b);
• Regarding stimulation pulses: maximum pulse width stated at all the research works on this field is 2 ms;
• Regarding sensitive components, place them into a Faraday cage for shielding against high-frequency noise (Yu et al., 2011a).

For the particular requirements for the basic safety and essential performance of MR diagnostic equipment, IEC 60601-2-33 should be followed; it also establishes the responsibility of the research organization in employing external devices in the scanner room (IEC-60601-2-33-1, 2013).

At this stage, whether possible, the components of the device can be altered to suppress any failed assessment mentioned above (repeating the safety assessment). Yu et al. (2011a) proposes a set of considerations about design principles and Schenck (1996) performed a detailed study on the physical properties of materials that can be useful here. If the device passes all the aforementioned tests, it should be tested for compatibility.

#### 4.3.2. Compatibility assessment

Regarding image quality assessment, we propose a set of metrics to measure the main noise sources potentially introduced by the device. These methods are ordered by decreasing specificity to noise sources and increasing experimental complexity and duration (Farrens et al., 2018):

• First, we propose the measurement of the RF emissions of the device (if applicable) and the eventual interference/coupling with the RF pulses of the MR scanner [RF-interferences (RFI)]; to do so, a standard quality assurance test (provided as a utility in commercial MRI scanners) can be used [for more details please consult the work of Farrens et al. (2018)];
• Additionally, we propose the visual inspection of the acquired images and quantification (using a spatial quality measure, also known as a static test) of their impact on the acquired images (if applicable). Static tests include the calculation of SNR, spatial homogeneity, image distortion or image non-uniformity, and others. NEMA provides several methods for measuring the SNR (NEMA MS 1, 2020) and spatial homogeneity (NEMA MS 3, 2020). Here, specific sequences for magnetic field mapping or the calculation (using the Biot-Savart law) of the changes in the magnetic fields produced by the device could also be performed to verify the impact of magnetic fields generated by the electric currents running in active devices (Hartwig et al., 2017);
• Then, dynamic tests to analyze whether the deviation of the signal of a voxel over time is affected by the stimulation device used during imaging should be performed (Farrens et al., 2018). Dynamic tests include the calculation of temporal stability measures, such as the tSNR, SFNR, or standard deviation of the image intensity time-course; there are several approaches to calculate these metrics, although no official standard is available [for details see the works of Friedman and Glover (2006), Gassert et al. (2006a), Hartwig et al. (2017), Farrens et al. (2018)]. Low tSNR values are expected since they will enable the detection of signal changes that arise from task-related fMRI data, whose variability is in the order of 1% change in magnitude (Farrens et al., 2018).

Image compatibility measures must be compared with the device outside the scanner room (reference), with the device turned off in the scanner room (silent device), with the device turned on in the scanner room (power device), and with the device turning on and operating (functioning device). The reference measure should be repeated at the end of the experiment in order to verify the repeatability of the measures and their insensitivity to the low-frequency thermal drift of the scanner (Hartwig et al., 2017). To check whether the calculated metrics (repeated for each condition suggested previously) have been “significantly” changed, standard statistical tests should be employed.

Regarding device performance within the MR environment, this should be quantitatively compared with the performance outside the MR environment using specific tests, according to the devices’ actuation principles and characteristics. This is of utmost importance for electric devices since disturbance of sensors, electronics, active components, and deterioration of device function can occur. Yu et al. (2011a) proposes a measure to evaluate the desired function of a device, comparing its performance inside and outside the MR environment; the authors also propose methodologies to assess the disturbance of sensors, electronics, and active components (specific for electronic devices).

The aforementioned tests should be performed on each component of the device first and only then at the final prototype, including at least all device orientations relative to B0, all slices and phase encoding directions, and further specific sequence adjustments (Schaefers and Melzer, 2006). Likewise, these tests should be performed both in phantoms (simulating the human region to be imaged) and human participants, as well as using both anatomical/structural and functional acquisition sequences; ideally, compatibility assessment should be performed using the sequences that will be applied in the future during real acquisitions with participants.

Additional compatibility assessments should be performed according to the characteristics of each device. Once the device has passed the aforementioned tests, it could be assessed for participant acceptability.

#### 4.3.3. Participant acceptability

User acceptability tests must assess the usability of the device and end-user experience; we suggest a qualitative assessment, first outside the scanner and then inside, comparing both. Supplementary Table 3 contains a set of questions that could be used for this qualitative assessment (Supplementary Table 3). Lastly, but not mandatory, pilot tests on human subjects should also be carried out to verify the reliability of the fMRI study in terms of brain activity.

#### 4.3.4. Final considerations

The proposed protocol might be time- and resource-consuming. Researchers are responsible for selecting the most critical assessments according to their device. However, safety must never be neglected because it could represent danger for participants, researchers, and/or MR-technologists. Therefore, we advise researchers to perform all of the safety assessments proposed in section “4.3.1. Safety assessment” (whenever justified by components/actuation principle of the device). Additionally, it is also strictly necessaire to verify the correct functioning of the device inside the MR room as compared to outside.

Concluding, safety, compatibility, and acceptability assessments (and other eventual relevant assessments) depend (in general) on the materials and design of the device as well as the MR environment. The development of somatosensory stimulation devices to be used in the MR environment is a complex task and demands knowledge of different fields, thus collaboration between engineers, physicists, clinicians, and other technical personnel is important. Since MR technology is in progress, several new technological developments are expected (e.g., increasing B0); in this way, the complexity of testing devices for safety and compatibility in the MR environment is expected to increase even more. Labeling a device as MR-safe/MR-compatible is always dependent on the experimental conditions (e.g., magnitude of B0, etc.). Whenever these conditions change, the device must be tested again.

## 5. Conclusion

This systematic review provides a comprehensive overview of what has been done in the field of MR compatibility and the main issues of published studies, focused on somatosensory stimulation devices for research purposes. It also provides background information about international standards that must be followed for any researcher interested in the design and development of devices to be used in the MR environment (independent of the intended application), but more generally to commercial product development teams and the technical leadership of MRI brain research facilities. Future studies could use this review as a technical guide, focusing their work on clear frameworks following the proposed protocol and the international standards presented. Special attention should also be taken to the reporting of the methodological description of the assessments performed.

## Data availability statement

The original contributions presented in this study are included in the article/Supplementary material, further inquiries can be directed to the corresponding author.

## Author contributions

CT, AS, BD, JP, TS, and MC-B conceived and design the study. CT performed the initial search for relevant manuscript, screened the title and abstract of the records returned, and wrote the manuscript. CT, AS, BD, JP, and TS reviewed the manuscript selected to assess the fulfillment of the eligibility criteria, voted on the manuscript to include in this review, and summarized the full text of the included manuscript. AS, BD, JP, TS, and MC-B reviewed the manuscript. All authors read and approved the final manuscript.

## Footnotes

1 According to the standards of the American Society for Testing and Materials (ASTM International), the MR environment corresponds to the area within the 5-gauss line of an MR system. The American College of Radiology (ACR) guidance document on MR safe practices states that this line delimits zone IV, the room where the magnet is located (ACR Committee on MR Safety, 2020); access to this zone should be highly controlled.

2
http://www.ncbi.nlm.nih.gov/pubmed

3
https://www.elsevier.com/solutions/scopus

4
https://webofknowledge.com/

5 Version accessed: ASTM F2052-02 (2002).

6 Version accessed: ASTM F2213-04 (2004).

7 Version accessed: ASTM F2182-02 (2002).
