# Corrigendum: Assessing MR-compatibility of somatosensory stimulation devices: a systematic review on testing methodologies

**DOI:** 10.3389/fnins.2024.1423928

**Published:** 2024-05-09

**Authors:** Carolina Travassos, Alexandre Sayal, Bruno Direito, João Pereira, Teresa Sousa, Miguel Castelo-Branco

**Affiliations:** ^1^Coimbra Institute for Biomedical Imaging and Translational Research (CIBIT), University of Coimbra (UC), Coimbra, Portugal; ^2^Siemens Healthineers AG, Lisbon, Portugal; ^3^Institute for Nuclear Sciences Applied to Health (ICNAS), University of Coimbra (UC), Coimbra, Portugal; ^4^Instituto do Ambiente, Tecnologia e Vida (IATV), Coimbra, Portugal; ^5^Faculty of Medicine (FMUC), University of Coimbra (UC), Coimbra, Portugal

**Keywords:** magnetic resonance imaging (MRI), functional MRI (fMRI), somatosensory stimulation devices, compatibility, safety, MR-compatible, MR-safe

In the published article, the Funding statement was missing. The correct Funding statement appears below.

“The authors would like to acknowledge the support by The Portuguese Foundation for Science and Technology (FCT) through the projects FCT/UIDB/4950/Base/2020 and FCT/UIDP/4950/Programatico/2020.”

The authors apologize for this error and state that this does not change the scientific conclusions of the article in any way. The original article has been updated.

