# Supplementary material for: Assessing MR-compatibility of somatosensory stimulation devices: A systematic review on testing methodologies
Source: Front Neurosci. 2023 Jan 26;17:1071749. doi: 10.3389/fnins.2023.1071749 (PMC9909190; doi:10.3389/fnins.2023.1071749)
Supplement: Supplementary file 1 [file Data_Sheet_1.PDF]

Supplementary material

Table S1 - Main known hazards for somatosensory stimulation devices and corresponding assessment methodologies according to international standards. *(Abbreviations: ASTM-American Society for Testing and Materials; ISO/TS-International Organization for Standardization-Technical standard; NEMA-National Electrical Manufacturers Association; IEC- International Electrotechnical Commission; RF-radiofrequency; MRI-magnetic resonance imaging; SAR-specific absorption rate; SNR-signal-to-noise ratio; tSNR-temporal signal-to-noise ratio).* (Delfino and Woods, 2016; Hartwig et al., 2017; U.S. Food and Drug Administration, 2021).

	Topic	Test description	Standard	Application
**SAFETY**	Magnetically Induced Displacement Force	Standard test method for measurement of magnetically induced displacement force on medical devices in the magnetic resonance environment	ASTM F2052	Passive implantable and non-implantable medical devices
		B0-induced force	ISO/TS 10974	Active implantable medical devices
	Magnetically Induced Torque	Standard test method for measurement of Magnetically induced torque on medical devices in the magnetic resonance environment	ASTM F2213	Passive implantable and non-implantable medical devices
		B0-induced torque	ISO/TS 10974	Active implantable medical devices
	Heating	Standard test method for measurement of RF-induced heating near passive implants during MRI	ASTM F2182	Passive implantable medical devices
		Gradient field-induced heating	ISO/TS 10974	Active implantable medical devices
		RF-field-induced heating		
	Vibration	Gradient field-induced vibration		
	Extrinsic electrical potential	Gradient field-induced lead voltage		
	Rectification	RF field-induced rectified lead voltage		
	Device malfunction	Static magnetic field-induced device malfunction		

		Gradient field-induced device malfunction		
		RF field-induced device malfunction		
	SAR	Characterization of the SAR for MRI systems	NEMA MS8-2008	Any type of device
		Determination of local SAR in diagnostic MRI	NEMA MS10-2008	
**COMPATIBILITY**	Image quality	Standard test method for Evaluation of MR Image Artifacts from Passive Implants	ASTM F2119	Passive/active implantable medical devices
		Image artifact evaluation	ISO/TS 10974	Active implantable medical devices
	SNR/tSNR	The standard for determination SNR in diagnostic MRI	NEMA MS1-2008	Any type of device
	Image uniformity	Determination of image uniformity in diagnostic MRI	NEMA MS3-2008	Any type of device
**OTHER**	General requirements	General requirements for basic safety and essential performance	IEC 60601-1	Medical electrical equipment
	General requirements for safety	Collateral standard: Electromagnetic compatibility - Requirements and tests	IEC 60601-1-2	Medical electrical equipment
	MR scanner	Particular requirements for the basic safety and essential performance of magnetic resonance equipment for medical diagnosis	IEC 60601-2-33	MR scanners
	Labelling/Icons	Standard Practice for Marking Medical Devices and Other Items for Safety in the Magnetic Resonance Environment	ASTM F2503	Medical devices

Table S2 – Main properties of records included in the systematic review regarding 1) device characteristics (typology, intended application, and design), 2) MR-scanner characteristics (scanner type and sequences), and 3) safety, compatibility, and user acceptability tests performed and corresponding results and conclusions about acceptability. *(Abbreviations: DC - direct current; N.A. - Information not available; NEMA - National Electrical Manufacturers Association; MR – Magnetic resonance; SNR – Signal to noise ratio; SD – standard deviation; RF – radiofrequency; ROI - Region-of-interest; SFNR - Signal-to-fluctuation-noise ratio; tSNR: temporal signal to noise ratio).*

<table>
<tr><th>Title</th><th colspan="5">An MRI-compatible hand sensory vibrotactile system (Wang et al., 2015)</th></tr>
<tr><th>Device typology</th><th>Intended application</th><th colspan="2">Device design</th><th>MR scanner</th><th>MR sequences</th></tr>
<tr><td>Piezoelectric</td><td>Hands</td><td colspan="2">Outside the MR room: Noise source and noise amplifier.
Inside the MR room: piezoelectric vibrators, leads, battery, and amplifier.
Communication: penetration panel (BNC connector and coaxial wires).</td><td>N.A.</td><td>Anatomic</td></tr>
<tr><td rowspan="2">Safety</td><td colspan="2">Tests</td><td colspan="3">Outside the MR room: attraction force tested with a strong magnet (BNC connector and vibrators);
Inside the MR room: projectile effect, conductance, and heating (BNC connector and vibrators).</td></tr>
<tr><td colspan="2">Results</td><td colspan="3">Outside the MR room: BNC connector was affected by the magnet however since it will be positioned outside the 10-gauss line, it is recommended to take care whilst installing;
Inside the MR room: no detectable forces or magnetic effects.</td></tr>
<tr><td rowspan="6">Compatibility</td><td rowspan="3">Image quality</td><td>Tests</td><td colspan="3">Image quality (no tests specified).</td></tr>
<tr><td>Conditions</td><td colspan="3">Phantom. 1. without the device in MR room; 2. device turned off; 3. device turned on and operating.</td></tr>
<tr><td>Results</td><td colspan="3">No signs of the device interfering with the MRI scans.</td></tr>
<tr><td rowspan="3">Device performance</td><td>Tests</td><td colspan="3">Power spectral density; Amplifier gain; Voltage fed to the vibrator; Vibration level.</td></tr>
<tr><td>Conditions</td><td colspan="3">Phantom. 1. without the device in MR room; 2. device turned off; 3. device turned on and operating.
Participant.</td></tr>
<tr><td>Results</td><td colspan="3">Power spectral intensity: similar when MRI is on and off;
Vibration level: not affected by the MRI scans.</td></tr>
<tr><td rowspan="3">User acceptability</td><td colspan="2">Tests</td><td colspan="3">Electrical shock and skin burning.</td></tr>
<tr><td colspan="2">Conditions</td><td colspan="3">Participant.</td></tr>
<tr><td colspan="2">Results</td><td colspan="3">No hazard of electric shock or risk of skin burning.</td></tr>
</table>

<table>
<tr><th>Title</th><th colspan="4">Development of a simple MR-compatible vibrotactile stimulator using a planar-coil-type actuator (Kim et al., 2013)</th></tr>
<tr><th>Device typology</th><th>Intended application</th><th>Device design</th><th>MR scanner</th><th>MR sequences</th></tr>
<tr><td>Electromagnetic</td><td>Hands</td><td>Outside the MR room: control unit;
Inside the MR room: drive unit and planar-coil actuator;</td><td>3T Magnetom Trio (Siemens)</td><td>Functional</td></tr>
</table>

		**Communication:** waveguide (optic cable).		
Safety	Tests		N.A.	
Compatibility	***Image quality***	Tests	SNR (no information about the method) and visual inspection.	
		Conditions	Phantom. 1. without the device in MR room; 2. with the device turned off, positioned 30cm away from the head coil; 3. with the device turned on and operating, in the same position.	
		Results	The difference in the SNRs was very small and there was no distortion of the MR images.	
	Device performance	Tests	Performance of the filter trap;<br>Measurement of stimulation signals from the planar-coil-type actuator with an accelerometer;<br>Stimulation intensity changes due to frequency changes.	
		Conditions	Phantom.	
		Results	Performance of the filter trap: device can be used without any compensation for all MR scanners below 3.0 T;<br>Measurement of stimulation signals from the planar-coil-type actuator: since the static magnetic field is in the direction of the y-axis and current is in the direction of the x-axis, the maximum force is induced in the direction of the z-axis, which is orthogonal to the x–y plane;<br>Stimulation intensity changes: almost constant with changing stimulation frequencies.	
User acceptability	Tests		N.A.	
	Conditions		N.A.	

Title	***A compatible electrocutaneous display for functional magnetic resonance imaging application*** *(Hartwig et al., 2006)*			
Device typology	***Intended application***	***Device design***	***MR scanner***	***MR sequences***
Electric	Hands/Fingers	**Outside the MR room:** main stimulator;<br>**Inside the MR room:** stimulator pads (with electrodes);<br>**Communication:** waveguide (coaxial cables).	1.5T Signa Horizon (GE)	Anatomic + Functional
Safety	Tests		N.A.	
Compatibility	***Image quality***	Tests	SNR (calculated according to the work of Sijbers et al. (1998));<br>SD of the image intensity time course, and visual inspection.<br>**Statistical analysis:** t and z tests.	
		Conditions	Phantom. 1. without the device in MR room; 2. with the device turned off; 3. with the device turned on and operating.	
		Results	The compatibility of the device in the MR–environment and the absence of unexpected and dangerous effects were proved. Conclusion - device passes the tests.	
	Device performance	Tests	N.A.	
		Conditions	N.A.	

<table>
<tr><td rowspan="3">User acceptability</td><td>Tests</td><td>Psychophysical laboratory tests.</td></tr>
<tr><td>Conditions</td><td>Participants were stimulated by an electric current with different parameters (amplitude and frequency) to determine the perceived tactile sensation (outside the MR room).</td></tr>
<tr><td>Results</td><td>Sensations described: slipping, vibration sensation, and pin-prick-sensation.</td></tr>
<tr><td>References:</td><td colspan="2">Sijbers, J., Den Dekker, A. J., Van Audekerke, J., Verhoye, M., and Van Dyck, D. (1998) Estimation of the noise in magnitude MR images, Magnetic Resonance Imaging, 16(1), 87-90.</td></tr>
</table>

<table>
<tr><td>Title</td><td colspan="5">Novel MRI-compatible tactile stimulator for cortical mapping of foot sole pressure stimuli with fMRI (Hao et al., 2013)</td></tr>
<tr><td>Device typology</td><td>Intended application</td><td colspan="2">Device design</td><td>MR scanner</td><td>MR sequences</td></tr>
<tr><td>Pneumatic</td><td>Foot</td><td colspan="2">Outside the MR room: air-compressor, control unit;
Inside the MR room: pneumatic actuator; support platform;
Communication: waveguide (air tubes).</td><td>3T Signa Excite HD (GE)</td><td>Anatomical + Functional + Field map</td></tr>
<tr><td>Safety</td><td colspan="2">Tests</td><td colspan="3">Not applicable.</td></tr>
<tr><td rowspan="6">Compatibility</td><td rowspan="3">Image quality</td><td>Tests</td><td colspan="3">SNR (anatomical images): calculated according to NEMA MS1 (2020) methods;
SFNR (functional images): calculated according to the method of Friedman and Glover (2006) - creation of a “signal image” from the voxel-wise time-series mean without the first 10 measurements; then, create a fluctuation noise image by detrending the voxel-wise time series with a second-order Legendre polynomial and computing the voxel-wise time-series standard deviation; divide the signal image by the noise image, and the SFNR is the ROI mean (and standard deviation) of the resulting SFNR image.
Mean and SD of the ROI from field maps.
Visual inspection.</td></tr>
<tr><td>Conditions</td><td colspan="3">Phantom. 1. without the device in the MR room; 2. with the device turned off; 3. with the device turned on and operating. All tests were calculated from a central 30-mm radius circular ROI in the center of the phantom.</td></tr>
<tr><td>Results</td><td colspan="3">All measures were virtually identical with the stimulation system present (whether powered on or off) as compared to the stimulation system absent.</td></tr>
<tr><td rowspan="3">Device performance</td><td>Tests</td><td colspan="3">Calibration: determine the relation between voltage input to the proportional electropneumatic valve on the air compressor and force output of the actuator, using an instrumented foot pressure insole.
Performance: measure the actual force produced on the foot sole in response to preprogrammed patterns of stimulation.</td></tr>
<tr><td>Conditions</td><td colspan="3">Participants; Inside/outside the scanner.</td></tr>
<tr><td>Results</td><td colspan="3">Calibration: Increasing the frequency of oscillation did not affect peak forces outside the magnet room, yet resulted in a small but significant reduction (1.58 N) in peak force production inside the magnet room.
Performance: capable of greater force output, as well as independent control of the force waveform and surface area over which pressure stimulation is applied.</td></tr>
</table>

User acceptability	Tests	N.A.
	Conditions	N.A.
References:	NEMA MS 1 (2020). NEMA Standards Publication MS 1-2008 (R2014, R2020). Standard for Determination of Signal-to-Noise Ratio (SNR) in Diagnostic Magnetic Resonance Imaging. Technical report, National Electrical Manufacturers Association (NEMA).<br>Friedman L, and Glover GH (2006) Report on a multicenter fMRI quality assurance protocol. *J Magn Reson Imaging.*23:827–839.	

Title	***Contact Force- and Amplitude-Controllable Vibrating Probe for Somatosensory Mapping of Plantar Afferences With fMRI*** *(Gallasch et al., 2006)*				
Device typology	***Intended application***	***Device design***		***MR scanner***	***MR sequences***
Electromagnetic	Foot	**Outside the MR room:** remote in/out unit; computer;<br>**Inside the MR room:** platform with foot supports; two vibration probes;<br>**Communication:** N.A.		1.5T Magnetom Sonata (Siemens) and 1.5 T Gyroscan (Phillips)	Functional
Safety	Tests		Measure the minimal distance for the safe operation of the vibration probes (corresponding to the 20-mT line/zone 4, according to guidelines from GE Medical Systems (1997) and others (Chinzei et al., 1999; Shellock, 2002; Kwong, 1995)) using a gaussmeter (measure the magnetic field along the z-axis).		
	Results		Device can be operated behind the 20-mT line.		
Compatibility	***Image quality***	Tests	RF emissions.		
		Conditions	Phantom. 1. with the device turned on but not operating; 2. with the device operating.		
		Results	RF emissions were a source of elevated noise so additional shielding and grounding of the cables were used to reduce it, resulting in an imaging quality similar to that obtained with the stimulator outside of the MR room. Additionally, the authors reported that the use of analog electronics and limiting the control bandwidth (0–1 kHz) also contributed to keeping the effects on imaging small.		
	Device performance	Tests	N.A.		
		Conditions	N.A.		
User acceptability	Tests		N.A.		
	Conditions		N.A.		
References:	GE Medical Systems. MR safety and MR compatibility. http://www.ge.com/medical/mr/iomri/safety.htm; 1997.<br>Chinzei K, Kikinis R, and Jolesz F. (1999) MR compatibility of mechatronic devices, design criteria. *Proceedings of the MICCA 99. Lecture Notes Comput Sci*.1679:1020 –1031.<br>Shellock FG. (2002) Magnetic resonance safety update 2002, implants and devices. *J Magn Reson Imaging*;16:485–496.<br>Kwong KK. (1995) Functional magnetic resonance imaging with echo-planar imaging. *Magn Reson Q*. 11:1–20.				

<table>
<tr><td>Title</td><td colspan="5">Neuroscience robotics to investigate multisensory integration and bodily awareness (Duenas et al., 2011)</td></tr>
<tr><td>Device typology</td><td>Intended application</td><td colspan="2">Device design</td><td>MR scanner</td><td>MR sequences</td></tr>
<tr><td>Piezoelectric</td><td>Legs/Back</td><td colspan="2">Outside the MR room: computer, power, control box;
Inside the MR room: ultrasonic motors, stroking modules;
Communication: penetration panel (shielded cables and radio frequency filters) and waveguide (optical fiber).</td><td>3T Trio and 7T Magnetom (Siemens)</td><td>Field-map</td></tr>
<tr><td rowspan="2">Safety</td><td colspan="2">Tests</td><td colspan="3">Not applicable.</td></tr>
<tr><td colspan="2">Additional safety measures</td><td colspan="3">Emergency switch at the control room.</td></tr>
<tr><td rowspan="5">Compatibility</td><td rowspan="3">Image quality</td><td>Tests</td><td colspan="3">SNR (no information about the method) and image disturbances/shifts.
Statistical analysis: two-sample t-test.</td></tr>
<tr><td>Conditions</td><td colspan="3">Phantom. 1. with the device turned off; 2. with the device operating.</td></tr>
<tr><td>Results</td><td colspan="3">No significant difference between the SNR time series of the two conditions ($p=0.56$).</td></tr>
<tr><td rowspan="2">Device performance</td><td>Tests</td><td colspan="3">N.A.</td></tr>
<tr><td>Conditions</td><td colspan="3">N.A.</td></tr>
<tr><td rowspan="2">User acceptability</td><td colspan="2">Tests</td><td colspan="3">N.A.</td></tr>
<tr><td colspan="2">Conditions</td><td colspan="3">N.A.</td></tr>
</table>

<table>
<tr><td>Title</td><td colspan="5">A new device for tactile stimulation during fMRI (Dresel et al., 2008)</td></tr>
<tr><td>Device typology</td><td>Intended application</td><td colspan="2">Device design</td><td>MR scanner</td><td>MR sequences</td></tr>
<tr><td>Pneumatic</td><td>Face/Hand</td><td colspan="2">Outside the MR room: control unit and signal converter;
Inside the MR room: driving unit, Bowden wires, positioning units, and Von-Frey filaments;
Communication: penetration panel (shielded cable).</td><td>1.5T Symphony (Siemens)</td><td>Anatomical + Functional</td></tr>
<tr><td>Safety</td><td colspan="2">Tests</td><td colspan="3">N.A.</td></tr>
<tr><td rowspan="5">Compatibility</td><td rowspan="3">Image quality</td><td>Tests</td><td colspan="3">Image differences i.e., subtract corresponding images of the same and of different series from each other as such subtraction images are rather sensitive to image distortions and other imaging artifacts.</td></tr>
<tr><td>Conditions</td><td colspan="3">Phantom; 1. without the device in the MR room; 2. with the device turned off; 3. with the device turned on and operating.</td></tr>
<tr><td>Results</td><td colspan="3">No differences in image-by-image comparisons apart from the inherent scanner noise.</td></tr>
<tr><td rowspan="2">Device performance</td><td>Tests</td><td colspan="3">N.A.</td></tr>
<tr><td>Conditions</td><td colspan="3">N.A.</td></tr>
</table>

User acceptability	Tests	N.A.
	Conditions	N.A.

Title	***A haptic force feedback device for virtual reality-fMRI experiments*** *(Di Diodato et al., 2007)*			
Device typology	***Intended application***	***Device design***	***MR scanner***	***MR sequences***
Piezoelectric	Upper limbs	**Outside the MR room:** computer and power;<br>**Inside the MR room:** piezoelectric motor, fiber optic position tracking device, and fMRI-compatible goggles;<br>**Communication:** penetration panel (shielded cable).	3T	Anatomical + Functional
Safety	Tests		Not applicable.	
Compatibility	***Image quality***	Tests	SNR and SFNR: calculated according to the method presented in (Glover and Lai, 1998).<br>Visual inspection (signal loss, spatial distortions, and other image artifacts).<br>**Statistical analysis:** 1-factor ANOVA	
		Conditions	Phantom. 1) without the device in MR room; 2) with the device turned off; 3) with the device turned on; 4) with the device turned on and operating.	
		Results	ANOVA results showed no significant effect of introducing the device into the fMRI environment.<br>Qualitative observations confirmed that the device functioned fully during fMRI, and without causing spatial distortions, signal loss, or blurring in the images.	
	Device performance	Tests	Haptic performance: maximum forces applied; interaction bandwidth; transmission delay.	
		Conditions	Results were compared to ideal and current standards.	
		Results	The prototype exhibited haptic performance parameters that fall within the ideal range and the typical ranges exhibited by other (non-fMRI-compatible) haptic devices.	
User acceptability	Tests		N.A.	
	Conditions		N.A.	
References:	Glover, G. H., and Lai, S. (1998) *Self-navigated spiral fMRI: Interleaved versus single-shot.* Magn. Reason. Med. 39(3), 361-368.			

Title	***Novel magnetomechanical MR compatible vibrational device for producing kinesthetic illusion during fMRI*** *(Carr et al., 2013)*			
Device typology	***Intended application***	***Device design***	***MR scanner***	***MR sequences***
Electromagnetic	Upper limbs	**Outside the MR room:** computer;<br>**Inside the MR room:** coil, position frame, and optoisolator;<br>**Communication:** Waveguide (fiber optic).	3T Magnetom Verio (Siemens)	Anatomical + Functional
Safety	Tests		N.A.	

Compatibility	***Image quality***	Tests	tSNR (no information about the method) and visual inspection.
		Conditions	Phantom. 1) without the device in MR room; 2) with the device turned off; 3) with the device turned on; 4) with the device turned on and operating.
		Results	The authors reported that the device operated safely in the MR environment, without degradation to the tSNR and images were free of artifacts. The small electromagnetic field created by the current flowing through the coil was not sufficient to interfere with the magnetic field of the scanner or its operation.
	Device performance	Tests	N.A.
		Conditions	N.A.
User acceptability	Tests		N.A.
	Conditions		N.A.

Title	***Development of a magnetic resonance-compatible tactile orientation delivery system*** *(Chen et al., 2014)*			
Device typology	***Intended application***	***Device design***	***MR scanner***	***MR sequences***
Piezoelectric	Upper limbs	**Outside the MR room:** computer and controller;<br>**Inside the MR room:** ultrasonic motor, J.V.P. domes, and support;<br>**Communication:** N.A.	3T	Functional
Safety	Tests	Not applicable.		
Compatibility	***Image quality***	Tests	SNR, according to the formula: $SNR = \frac{\left(2-\frac{\pi}{2}\right)^{\frac{1}{2}}\times S_p}{N_{air}}$, S_p represents the average signal value in the ROI in the phantom and N_{air} represents the SD of the signal value in the ROI outside of the phantom (radius of the ROI is 15mm)	
		Conditions	Phantom. Assessments were performed with/without shielding over the ultrasonic motor and the device at 2 different positions (the authors did not specify).	
		Results	The SNR values were 83.6% and 22.6% for each position. The authors did not make a comment on these values.	
	Device performance	Tests	N.A.	
		Conditions	N.A.	
User acceptability	Tests	N.A.		
	Conditions	N.A.		

Title	***Active mechatronic interface for haptic perception studies with functional magnetic resonance imaging: Compatibility and design criteria*** *(Gassert et al., 2006a)*

<table>
<tr><th>Device typology</th><th>Intended application</th><th colspan="2">Device design</th><th>MR scanner</th><th>MR sequences</th></tr>
<tr><td>Piezoelectric</td><td>Fingers</td><td colspan="2">Outside the MR room: computer;
Inside the MR room: ultrasonic motor, tubes/pipes, and linear potentiometer;
Communication: waveguide.</td><td>1.5T Signa CV/i (GE)</td><td>Anatomical + Functional</td></tr>
<tr><td rowspan="2">Safety</td><td colspan="2">Tests</td><td colspan="3">Not applicable
(the goal of the paper was to test the compatibility of the materials and components for device development).</td></tr>
<tr><td colspan="2">Additional safety measures</td><td colspan="3">Emergency buttons, software security routines, mechanical end-of-travel limitations, and electrical end-of-travel switches.</td></tr>
<tr><td rowspan="3">Compatibility</td><td rowspan="3">Image quality</td><td>Tests</td><td colspan="3">SNR, according to the formula: $SNR = \frac{P_{center}}{\left(\frac{1.53}{4}\right)\sum_{i=1}^{4} SD_i}$, P_{center} is the mean value of a 10x10 voxel area at the center of the image, and SD_i is the mean SD of the ith of four 5x5 voxel areas in the corners of the image; this operation was repeated for each image, so 20 SNR estimates were computed for each sequence.
SD of the image intensity time course: calculated for each image sequence from a 15x15 voxel ROI located in the center of the phantom, resulting in 225 SD values for each image sequence.
Statistical analysis: SNR - unpaired t-test; SD of the image intensity time course - z test.</td></tr>
<tr><td>Conditions</td><td colspan="3">These tests were performed both during the design phase (to determine the compatibility of the individual components) and to test the final prototype: Phantom: 1) phantom only (reference); 2) phantom with each component/final prototype.
Each component was tested under different conditions:
- brass and aluminum pipes: positioned at the entrance of the scanner bore and moving along the z-axis, aligned with the patient bed and the static magnetic field;
- servo motors (DC motor and ultrasonic motor): off and working; DC motor will only be used outside the scanner room since it has ferromagnetic materials and permanent magnets;
- potentiometers: different materials (carbon and plastic).
The final prototype was tested with the slave module placed at the bore entrance on one side of the patient bed and the device working (ON).
Several baseline sequences were acquired at different times in the same session in order to examine whether they were affected by significant differences regarding system instabilities. Having verified that there was no relevant change among the baseline sequences, one baseline was randomly selected as a reference.</td></tr>
<tr><td>Results</td><td colspan="3">Results for each component:
- brass and aluminum pipes: no significant difference between the sequences with materials and the baseline sequences; both can be used in the hand region (entry of the scanner bore) or further away, both for static and moving components;
- servo motors: compatible both in off and working conditions;</td></tr>
</table>

			- potentiometer A showed significant differences to the baseline images, while linear potentiometer B did not produce significant changes.<br>Results for final prototype:<br>- The results show no significant difference between the image sequences with the running device and the baseline images.
	Device performance	Tests	Not applicable<br>(the goal of the paper was to test the compatibility of the materials and components for device development)
		Conditions	Not applicable
User acceptability	Tests		Not applicable
	Conditions		Not applicable

Title	***Development and Evaluation of a tactile speed stimulator for MRI environment*** *(Guo et al., 2012)*			
Device typology	***Intended application***	***Device design***	***MR scanner***	***MR sequences***
Piezoelectric	Upper limbs	**Outside the MR room:** computer and motor controller;<br>**Inside the MR room:** tactile stimulator, ultrasonic motors, and reaction key;<br>**Communication:** N.A.	3T (Siemens)	Anatomical + Functional
Safety	Tests		Not applicable.	
	Additional safety measures		Safety switch.	
Compatibility	***Image quality***	Tests	RF emission and SNR (using the air-signal method).	
		Conditions	Phantom.	
		Results	The digressive rate of the image with the device was less than 2.3%, indicating that almost no measurable modification of the SNR or RF noise was detected. No artifacts caused by the RF noise were found.	
	Device performance	Tests	N.A.	
		Conditions	N.A.	
User acceptability	Tests		N.A.	
	Conditions		N.A.	

Title	***Development of a Novel fMRI Compatible Stimulator System for Tactile Study*** *(Huang et al., 2017)*			
Device typology	***Intended application***	***Device design***	***MR scanner***	***MR sequences***
Pneumatic	Fingers	**Outside the MR room:** control box, air compressor, and computer;<br>**Inside the MR room:** tactile stimulus regulating apparatus;<br>**Communication:** waveguide (plastic tubes).	3T Prisma (Siemens)	Functional

<table>
<tr><td rowspan="1">Safety</td><td colspan="2">Tests</td><td>Not applicable.</td></tr>
<tr><td rowspan="5">Compatibility</td><td rowspan="3">Image quality</td><td>Tests</td><td>SNR, according to the formula: $SNR = \frac{\left(2-\frac{\pi}{2}\right)^{\frac{1}{2}} \times S_p}{N_{air}}$, N_{air} is the SD of outside noise and S_p is the mean of the signals in the phantom. Visual inspection.</td></tr>
<tr><td>Conditions</td><td>Phantom.</td></tr>
<tr><td>Results</td><td>The digressive rate of the image with the system was 1.84%. The SNR was 11.96 without the system and 11.74 with the system running. No artifacts caused by the RF noise were found.</td></tr>
<tr><td rowspan="2">Device performance</td><td>Tests</td><td>N.A.</td></tr>
<tr><td>Conditions</td><td>N.A.</td></tr>
<tr><td rowspan="2">User acceptability</td><td colspan="2">Tests</td><td>N.A.</td></tr>
<tr><td colspan="2">Conditions</td><td>N.A.</td></tr>
</table>

<table>
<tr><td>Title</td><td colspan="5">Development of a Dual Tactile Pattern Presentation Device using in MRI Environment (Yang et al., 2009)</td></tr>
<tr><td>Device typology</td><td>Intended application</td><td colspan="2">Device design</td><td>MR scanner</td><td>MR sequences</td></tr>
<tr><td>Piezoelectric</td><td>Hands</td><td colspan="2">Outside the MR room: computer;
Inside the MR room: disk for pattern presentation, ultrasonic motor, set of fingers position control units, and reaction keypad;
Communication: N.A.</td><td>3T Magneton (Siemens)</td><td>Anatomical + Functional</td></tr>
<tr><td rowspan="2">Safety</td><td colspan="2">Tests</td><td colspan="3">Not applicable.</td></tr>
<tr><td colspan="2">Additional safety measures</td><td colspan="3">Safety switch; insulation of electrical components; cables and motors contained in a plastic frame; the device does not preclude the safe exit of the participant.</td></tr>
<tr><td rowspan="5">Compatibility</td><td rowspan="3">Image quality</td><td>Tests</td><td colspan="3">SNR, according to the formula: $SNR = \frac{S_p}{\left(\frac{N_s}{\sqrt{2}}\right)}$, S_p is the average signal rate of ROI-1 or ROI-2 and N_s is the standard difference of both ROI-1 and ROI-2. ROI-1 and ROI-2 were defined at the center of the phantom.</td></tr>
<tr><td>Conditions</td><td colspan="3">Phantom. 1) without the device in MR room; 2) with the device turned on and operating</td></tr>
<tr><td>Results</td><td colspan="3">The SNR of the present study is lower than 5%. Therefore there was no measurable modification of the SNR in the MRI environment when operated with the main device.</td></tr>
<tr><td rowspan="2">Device performance</td><td>Tests</td><td colspan="3">N.A.</td></tr>
<tr><td>Conditions</td><td colspan="3">N.A.</td></tr>
<tr><td rowspan="2">User acceptability</td><td colspan="2">Tests</td><td colspan="3">N.A.</td></tr>
<tr><td colspan="2">Conditions</td><td colspan="3">N.A.</td></tr>
</table>

Title	***Development and Evaluation of a MRI-Compatible Tactile Orientation Stimulator*** *(Yu et al., 2011b)*			
Device typology	***Intended application***	***Device design***	***MR scanner***	***MR sequences***
Piezoelectric	Fingers	**Outside the MR room:** computer and motor controller;<br>**Inside the MR room:** J.V.P. domes, ultrasonic motors, and reaction key;<br>**Communication:** N.A.	3T (GE)	Anatomical + Functional
Safety	Tests		Not applicable.	
	Additional safety measures		Safety switch.	
Compatibility	***Image quality***	Tests	SNR (using the air-signal method).	
		Conditions	Phantom. 1) without the device in MR room; 2) with the device turned on and operating	
		Results	The mean of the signals in the phantom was 1194.1 without the device and 1166.8 with the device active. The digressive rate of the image with the device was less than 5%. This digressive rate indicated that almost no measurable modification of the SNR or RF noise was detected. No artifacts caused by the RF noise were found.	
	Device performance	Tests	N.A.	
		Conditions	N.A.	
User acceptability	Tests		N.A.	
	Conditions		N.A.	

Title	***Programmable tactile pattern presentations operational under MRI to investigate neural mechanisms of tactile shape discrimination*** *(Yang et al., 2011)*			
Device typology	***Intended application***	***Device design***	***MR scanner***	***MR sequences***
Piezoelectric	Hands	**Outside the MR room:** computer, motor controller, force sensors, and electronic amplifier unit;<br>**Inside the MR room:** primary device and reaction key;<br>**Communication:** penetration panel (shielded cable).	1.5T (Philips)	Functional
Safety	Tests		Not applicable.	
	Additional safety measures		Safety switch; safe exit of the participant.	
Compatibility	***Image quality***	Tests	RF emission and SNR (using the air-signal method (Ogura et al., 2007)): $SNR = \frac{\left(2-\frac{\pi}{2}\right)^{\frac{1}{2}} \times S_p}{N_{air}}$, S_p is the mean of the signals in the phantom and N_{air} is the SD of outside noise.	
		Conditions	Phantom. 1) without the device in MR room; 2) with the device turned on and operating	

		Results	The SNR was 230.03 without the device and 222.86 with the device active. The digressive rate of the image with the device was 3.23%. This digressive rate indicated that almost no measurable modification of the SNR or RF noise was detected. No artifacts caused by the RF noise were found.
	Device performance	Tests	N.A.
		Conditions	N.A.
User acceptability	Tests		N.A.
	Conditions		N.A.

Title	***A new vibrotactile stimulator for functional MRI*** *(Harrington et al., 2000)*					
Device typology	***Intended application***	***Device design***		***MR scanner***	***MR sequences***	
Piezoelectric	Upper limbs	**Outside the MR room:** power source;<br>**Inside the MR room:** piezoceramic wafer;<br>**Communication:** penetration panel (insulated coaxial wire).		1.5T Vision (Siemens)	Functional	
Safety	Tests		**Outside the MR room:** attraction force with a small magnet;<br>**Inside the MR room:** interaction between the magnetic field and the current on the coaxial wire; heating.			
	Results		**Outside the MR room:** The piezoelectric wafer did not produce a force in the presence of the small magnet so the authors considered it MRI-compatible. They also highlighted that the type of compatibility, first or second kind, is not known because the exact susceptibility of the wafer is not known. However, this distinction is not important as long as the wafer is placed away from the imaging plane so that it cannot cause any distortions in the image, such as in the case of this device.<br>**Inside the MR room:** To estimate the force on the wire, the authors assumed the wire made a 10° angle with B0 and the wire had no shielding; the current in the wire ranged from 0.2 mA to 0.27 mA. In this case, the force on the wire would be 0.7 N, which is comparable to the weight of an object with a mass of approximately 71 g. No noticeable increase in the temperature of the wire during the fMRI experiment was noticed.			
Compatibility	***Image quality***	Tests	The authors named the aforementioned safety tests as compatibility tests, however, we considered them as safety tests.			
		Conditions				
	Device performance	Tests	N.A.			
		Conditions	N.A.			
User acceptability	Tests		Qualitative assessment.			
	Conditions		Participants.			
	Results		The subjects did not report any negative effects other than a little surprise when the stimulator was initially turned on.			

<table>
<tr><td>Title</td><td colspan="5">A new vibrator to stimulate muscle proprioceptors in fMRI (Montant et al., 2009)</td></tr>
<tr><td>Device typology</td><td>Intended application</td><td colspan="2">Device design</td><td>MR scanner</td><td>MR sequences</td></tr>
<tr><td>Pneumatic</td><td>Hands</td><td colspan="2">Outside the MR room: computer, controller unit, and air compressor;
Inside the MR room: vibrator;
Communication: N.A.</td><td>3T Bruker (Medspec 30/80 advance)</td><td>Functional</td></tr>
<tr><td>Safety</td><td colspan="2">Tests</td><td colspan="3">Not applicable</td></tr>
<tr><td rowspan="6">Compatibility</td><td rowspan="3">Image quality</td><td>Tests</td><td colspan="3">SNR (mean fMRI signal of the phantom versus the SD of the fMRI signal outside the phantom).
Statistical analysis: t-test.</td></tr>
<tr><td>Conditions</td><td colspan="3">Phantom. 4 vibrators, 20cm away from the phantom, with 2 different percentages of the apertures of the flowmetric gates (50 and 100%)</td></tr>
<tr><td>Results</td><td colspan="3">Activating the vibrators had no impact on the signal/noise ratio (all P values > 0.10), independently of the percentage of the aperture of the flowmetric gates and the number of vibrators activated. These results show that the vibrators are fMRI compatible when placed 20 cm or more away from the head, which is the case in most experiments since the vibrators are likely to be strapped on the legs or arms.</td></tr>
<tr><td rowspan="3">Device performance</td><td>Tests</td><td colspan="3">Efficiency and reliability of the pneumatic vibrators.</td></tr>
<tr><td>Conditions</td><td colspan="3">Phantom. Inside and outside the scanner.</td></tr>
<tr><td>Results</td><td colspan="3">This device could generate vibrations from 40 to 100/120 Hz (100 Hz inside and 120 Hz outside the scanner), which is the appropriate frequency range for activating muscle spindles. The vibration frequency was slightly lower inside than outside the scanner, independently of the percentage of the aperture of the flowmetric gate. However, there was no significant interaction between the percentage of aperture and the environment (inside/outside the scanner) of the vibrator ($F(1,11)=2.62$, $P=0.14$).</td></tr>
<tr><td rowspan="2">User acceptability</td><td colspan="2">Tests</td><td colspan="3">N.A.</td></tr>
<tr><td colspan="2">Conditions</td><td colspan="3">N.A.</td></tr>
</table>

<table>
<tr><td>Title</td><td colspan="5">Test-Retest Reliability and Concurrent Validity of an fMRI-Compatible Pneumatic Vibrator to Stimulate Muscle Proprioceptors (Goossens et al., 2016)</td></tr>
<tr><td>Device typology</td><td>Intended application</td><td colspan="2">Device design</td><td>MR scanner</td><td>MR sequences</td></tr>
<tr><td>Pneumatic</td><td>Legs</td><td colspan="2">Outside the MR room: air compressor and computer;
Inside the MR room: main device;
Penetration panel: N.A.</td><td>3T Achieva (Philips)</td><td>Anatomical + Functional</td></tr>
<tr><td>Safety</td><td colspan="2">Tests</td><td colspan="3">Not applicable</td></tr>
</table>

Compatibility	***Image quality***	Tests	tSNR (no information about the method) and visual inspection.
		Conditions	Phantom. Phantom. 1) without the device in MR room; 2) with the device position of the lower back turned off; 3) with the device position of the lower back turned on; 4) with the device positioned 15cm from the phantom turned off; 5)4) with the device positioned 15cm from the phantom turned on.
		Results	The differences in mean tSNR values between the different phantom scan conditions were very small, confirming that the pneumatic muscle vibrators do not affect MRI image quality and thus are fMRI-compatible when placed 15 cm or more away from the head.
	Device performance	Tests	N.A.
		Conditions	N.A.
User acceptability	Tests		N.A.
	Conditions		N.A.

Table S3 - Qualitative assessment of the somatosensory stimulation device. Characterization of each topic inside and outside the MR environment.

Topics	Outside the MR environment	Inside the MR environment
**1. Quantify the intensity of the stimulation.**<br>Use a 0-10 point scale: 0 - means no perceptible sensation, and 10 - means unbearable sensation.		
**2. Describe the main sensation provoked by the stimulation.**<br>Choose between the following descriptors: scratching, knocking, stinging, muscle twitch, tickling, pinching, and itching. If none of these apply, please specify.		
**3. Quantify the pain provoked by the stimulation.**<br>Use a 0-10 point scale: 0 - means no pain, and 10 - means unbearable pain.		
**4. Specify the localization of the stimulation.**<br>Choose between the following locations: between electrodes, both electrodes, in a specific electrode (please specify), beyond the electrodes, or other (please specify).		
**5. Specify the beginning, ending, and duration of the stimulation.**<br>Compare if the beginning/end/duration of the sensation reported by the participant corresponds to the start/end/duration of stimulation, respectively.		
**6. Does the stimulation provoke any reaction in your skin?**<br>Look for signs of skin redness and erythema (or others) after stimulation.		
Overall opinion:<br>**According to your perception, the stimulation was equal in both environments?** If not, please describe the main differences.		
